# Combined transcriptome and metabolome analysis reveals the effects of light quality on maize hybrids

**DOI:** 10.1186/s12870-023-04059-4

**Published:** 2023-01-18

**Authors:** Weimin Zhan, Guanghui Guo, Lianhua Cui, Muhammad Abdul Rehman Rashid, Liangliang Jiang, Guanghua Sun, Jianping Yang, Yanpei Zhang

**Affiliations:** 1 State Key Laboratory of Wheat and Maize Crop Science, Collaborative Innovation Center of Henan Grain Crops, College of Agronomy, Henan Agricultural University, Zhengzhou, 450002 China; 2 State Key Laboratory of Crop Stress Adaptation and Improvement, College of Agriculture, Henan University, Kaifeng, 475004 China; 3 Department of Bioinformatics and Biotechnology, Government College University Faisalabad, Faisalabad, 38000 Pakistan

**Keywords:** Maize, Light quality, Differentially expressed pattern, Interaction network, Non-additivity

## Abstract

**Background:**

Heterosis, or hybrid vigor, refers to the phenotypic superiority of an F_1_ hybrid relative to its parents in terms of growth rate, biomass production, grain yield, and stress tolerance. Light is an energy source and main environmental cue with marked impacts on heterosis in plants. Research into the production applications and mechanism of heterosis has been conducted for over a century and a half, but little is known about the effect of light on plant heterosis.

**Results:**

In this study, an integrated transcriptome and metabolome analysis was performed using maize (*Zea mays* L.) inbred parents, B73 and Mo17, and their hybrids, B73 × Mo17 (BM) and Mo17 × B73 (MB), grown in darkness or under far-red, red, or blue light. Most differentially expressed genes (73.72–92.50%) and differentially accumulated metabolites (84.74–94.32%) exhibited non-additive effects in BM and MB hybrids. Gene Ontology analysis revealed that differential genes and metabolites were involved in glutathione transfer, carbohydrate transport, terpenoid biosynthesis, and photosynthesis. The darkness, far-red, red, and blue light treatments were all associated with phenylpropanoid–flavonoid biosynthesis by Weighted Gene Co-expression Network Analysis and Kyoto Encyclopedia of Genes and Genomes enrichment analysis. Five genes and seven metabolites related to phenylpropanoid–flavonoid biosynthesis pathway were identified as potential contributors to the interactions between maize heterosis and light conditions. Consistent with the strong mid-parent heterosis observed for metabolites, significant increases in both fresh and dry weights were found in the MB and BM hybrids compared with their inbred parents. Unexpectedly, increasing light intensity resulted in higher biomass heterosis in MB, but lower biomass heterosis in BM.

**Conclusions:**

The transcriptomic and metabolomic results provide unique insights into the effects of light quality on gene expression patterns and genotype–environment interactions, and have implications for gene mining of heterotic loci to improve maize production.

**Supplementary Information:**

The online version contains supplementary material available at 10.1186/s12870-023-04059-4.

## Background

Heterosis, also known as hybrid vigor, is a complex biological phenomenon that results in hybrid progeny with superior phenotypes, including growth rate, biomass production, grain yield, and stress tolerance [1–3]. For more than a century and a half, heterosis has been successfully used to improve crop yield and quality, and hybrid seeds have been used in nearly all maize production, > 70% of rice in China, > 70% of rye varieties in Europe, > 90% of rapeseed in Europe, and > 80% of cotton in India [4–8]. To further exploit the potential of heterosis in hybrids, heterosis mechanisms should be clarified.

Based on classical genetics, three main hypotheses have been proposed as the driving forces of heterosis [2, 9–11]. The “dominance” model attributes heterosis to the presence of superior dominant alleles in one of the two parental inbred lines, thereby complementing the deleterious recessive alleles in hybrids [12]. The dominant effects of two plant height genes (*qHT7.1* and *Dw3*) that exhibit repulsion linkage have been proposed to account for a significant amount of the heterosis in sorghum [13]. In contrast, the “overdominance” model attributes heterotic traits to allelic interactions at one or multiple loci. Examples of single overdominant genes responsible for yield heterosis are *SINGLE FLOWER TRUSS* (*SFT*) in tomato [14] and *HEADING DATE 3a* (*Hd3a*) in rice [15]. In addition, in the “epistasis” model heterosis is explained by genetic interactions of non-allelic loci [16, 17]. Due to its phenotypic and genetic complexity, the process of heterosis remains difficult to explain with these three main models.

In recent years, epigenetic variations, including small RNA, DNA methylation, and histone modifications, have been found to play important roles in the molecular mechanisms of hybrid vigor [18–22]. For example, *LATE ELONGATED HYPOCOTYL* (*LHY*) and *CIRCADIAN CLOCK-ASSOCIATED 1* (*CCA1*) were epigenetically altered in hybrids, resulting in increased photosynthesis, starch metabolism, bacterial defense, and biomass [23, 24]. Although significant progress has been made in clarifying certain aspects of heterosis, this process is underlain by complex interactions among genetic, epigenetic, and gene regulatory networks. Another layer of complexity in hybrid performance is the influence of environmental conditions, which further complicates the identification of heterosis-related genes.

As the driving force of photosynthesis, light is one of the most significant environmental cues regulating plant growth and reproduction in processes including seed germination, shade avoidance, disease resistance, and flowering time, and may also lead to heterosis [25, 26]. Sunlight is polychromatic, and the main wavelengths absorbed and monitored by plants are far-red light (700–750 nm), red light (600–700 nm), and blue light (400–500 nm) [27]. The genetic basis and molecular mechanisms underlying the impact of monochromatic light on maize hybrids and their parents require further investigation.

Here, transcriptomics and metabolomics data were generated from seedling shoots of the maize inbred parents B73 and Mo17, and their reciprocal F_1_ hybrids BM (B73 × Mo17) and MB (Mo17 × B73), raised in darkness (Dk) or under far-red (FR), red (R), or blue (B) light conditions. Notably, gene expression and metabolite abundance were significantly impacted by all four light conditions, resulting in distinct changes in the inbred parents and F_1_ hybrids. Gene Ontology (GO), Weighted Gene Co-expression Network Analysis (WGCNA), and Kyoto Encyclopedia of Genes and Genomes (KEGG) were utilized to identify unique and common interaction networks among the differentially expressed genes (DEGs) and differentially accumulated metabolites (DAMs) identified under different light conditions. In addition, biomass heterosis was investigated in terms of fresh and dry weights to clarify the interaction between maize heterosis and light. Taken together, the results elucidate genotype–environment interactions and have implications for gene mining of heterotic loci during maize production.

## Materials and methods

### Plant materials and light treatment

Maize inbred B73 and Mo17 were provided by the Institute of Crop Science, Chinese Academy of Agricultural Sciences. Two inbred lines were planted at the Henan Agricultural University farm (Zhengzhou, China) in the summer of 2019. Reciprocal-crosses and self-crosses were performed on the two parents. After harvesting, seeds of maize inbred parents (B73 and Mo17) and their F_1_ hybrids (B73 × Mo17, BM, and Mo17 × B73, MB) were grown in darkness at 26 °C for 6 days, and subsequently transferred to far-red (FR, 737 nm, 2.5 μmol m^−2^ s^−1^), red (R, 658 nm, 30.0 μmol m^−2^ s^−1^), or blue (B, 447 nm, 6.0 μmol m^−2^ s^−1^) light conditions, or kept in darkness for 24 h. After treatment, the seedling shoots from the four genotypes were sampled for RNA sequencing (RNA-seq) and metabolome analyses, using three and six biological replicates, respectively. Seedling tissues were immediately frozen in liquid nitrogen and stored at − 80 °C until further use. Seeds were soaked in sterile water for 2 days before planting to ensure consistent germination.

### RNA-seq library construction and illumina sequencing

Total RNA from 48 samples (4 treatments × 4 genotypes × 3 replicates) was extracted using the mirVana miRNA Isolation Kit (Ambion, Austin, TX, USA) according to the manufacturer’s instructions. RNA integrity was assessed with an Agilent 2100 Bioanalyzer (Agilent Technologies, Santa Clara, CA, USA). RNA samples with RNA integrity scores > 7.0 were used for further analysis. cDNA libraries were generated following the manufacturer’s protocol with the TruSeq Stranded mRNA LTSample Prep Kit (Illumina, San Diego, CA, USA). Libraries were sequenced on an Illumina HiSeq 2500 platform by OE Biotech Co., Ltd. (Shanghai, China).

### Transcriptome profiling

Raw sequence reads from each sample were processed using Trimmomatic [28] to generate high-quality trimmed reads. These clean reads were mapped to the maize reference genome (B73 RefGen_v4; http://ftp.ensemblgenomes.org/pub/plants/release-48/fasta/zea_mays/dna/) [29] using the HISAT2 program [30]. Gene expression levels were calculated and normalized to fragments per kilobase of transcript per million mapped reads (FPKM) values [31] using Cufflinks software [32]. HTSeq software [33] was utilized to obtain read counts for each gene. Pearson correlation coefficient (R^2^) values were calculated based on FPKM of each gene across biological replicates to assess the reliability of RNA-seq quantifications under each light condition. Principal component analysis (PCA) was performed using the FactoMineR R package. The DESeq2 R package [34] was used to standardize the data, exclude genes with < 10 counts per million reads. Benjaminiand Hochberg (BH) method was used for multiple correction [35]. DEGs were identified based on thresholds of fold change (FC) > 1.5 and false discovery rate (FDR) < 0.3.

### Metabolite extraction

Metabolites were extracted from six biological replicates for each of the four genotypes and four treatments according to previously described methods [36]. Briefly, 80 mg of seedling shoot was transferred to a 1.5 mL centrifuge tube containing two ball bearings. Then, 20 μL of 2-chloro-l-phenylalanine (0.3 mg/mL, dissolved in methanol) as an internal standard and 1 mL water and methanol mixture (3/7, v/v) were added to each sample. Samples that had been frozen at − 80 °C were ground at 60 Hz for 2 min, ultrasonicated at room temperature for 30 min, and centrifuged at 13,000 rpm at 4 °C for 10 min. Next, 300 μL of supernatant was dried in a freeze concentration centrifugal dryer. Then, 400 μL of water and methanol mixture (4/1, v/v) was added to each sample. Samples were vortexed for 30 s, held at 4 °C for 2 min, and then centrifuged at 13,000 rpm at 4 °C for 10 min. Finally, the supernatants were collected, filtered using a 0.22 μm microfilter, and transferred to a vial for liquid chromatography–mass spectrometry (LC–MS) analysis. The ACquity UPLC I-Class system and Vion IMS QTOF mass spectrometer (Waters Corporation, MA, USA) were used for metabolomics analysis performed by Shanghai Lu Ming Biological Technology Co., Ltd. (Shanghai, China).

### Metabolite data processing and normalization

The raw LC–MS data were collected using UNIFI 1.8.1 software and subjected to noise elimination, peak identification, retention time alignment, peak alignment, and normalization using Progenesis QI v2.3 software (Nonlinear Dynamics, Newcastle, UK), with the tolerance, fragment tolerance, and product ion threshold set to 5 ppm, 10 ppm, and 5%, respectively. Based on m/z values, secondary fragments, and isotope peaks, metabolites were identified and compared against the LipidMaps v2.3 (https://www.lipidmaps.org/), METLIN (http://metlin.scripps.edu), and KEGG (https://www.genome.jp/kegg/) databases. Metabolite quantification was performed according to previously described methods [36]. Orthogonal partial least-squares-discriminant analysis [37] and paired *t*-tests were used to identify DAMs. BH method was used for multiple correction [35]. DAMs were determined based on the thresholds of variable importance in projection (VIP) > 1 and FDR < 0.05.

### DEG and DAM analyses

DEGs and DAMs for pairwise comparisons between parental inbred lines and their hybrids were classified into 12 types based on previous study [38]. Types I and II showed that the expression level/ accumulation of DEGs and DAMs in F_1_ hybrids fell between the two parental inbred lines, and FCs of DEGs and DAMs between the parents and F_1_ hybrids were > 1.5. Types III and IV were characterized by DEGs and DAMs in F_1_ hybrids similar to those of the male parent, and different from those of the female parent (FC > 1.5). Types V and VI exhibited DEGs and DAMs in F_1_ hybrids similar to those of the female parent, and different from those of the male parent (FC > 1.5). Types VII, VIII, and IX contained DEGs and DAMs in F_1_ hybrids that were lower than either parent (FC > 1.5). Types X, XI, and XII contained DEGs and DAMs in F_1_ hybrids that were higher than either parent (FC > 1.5). The male parent had higher expression level/accumulation of DEGs and DAMs than the female parent in types VII and X (FC > 1.5). The parents had similar expression levels of DEGs or accumulation of DAMs in types VIII and XI. The female parent had higher expression levels or accumulation of DEGs and DAMs than the male parent in types IX and XII (FC > 1.5). Types I and II were considered additive categories; types III, IV, V, and VI were considered as “complete-incomplete dominance” categories; types VII, VIII, IX, X, XI, and XII represented overdominance. Complete-incomplete dominant and overdominant genes and metabolites are also known as non-additive genes and metabolites, respectively. UpSet and Venn plots of genes with significantly different expression in the F_1_ compared to the mid-parent value (F_1_-MPV DEGs) were generated using the UpSetR and VennDiagram R packages, respectively.

### WGCNA

Based on the FPKM values of genes and accumulation of metabolites, WGCNA of F_1_-MPV DEGs and DAMs was performed using the WGCNA R package [39]. The adjacency matrices of F_1_-MPV DEGs and DAMs were generated with soft threshold power β values of 18 and 17, respectively. The dynamic tree cut algorithm (mergeCutHeight = 0.25) was used for the hierarchical clustering. In addition, module–trait relationships were identified using two sets of binary variables, with parental inbred lines set to 0 and hybrids set to 1. A module was considered significant based on an absolute R^2^ > 0.6 and *P* < 0.05.

### GO and KEGG enrichment analyses

GO enrichment analysis of F_1_-MPV DEGs within the categories “molecular function” and “biological process” was performed using agriGO v2.0 (http://systemsbiology.cau.edu.cn/agriGOv2/) with singular enrichment analysis [40] and visualized in the “TreeMap” view of REVIGO (http://revigo.irb.hr/) [41]. Only GO terms with *P* < 0.05 are included in the main text. Both F_1_-MPV DEGs and DAMs were functionally annotated and mapped to KEGG pathways [42]. A pathway was considered significantly enriched at *P* < 0.01.

### Real-time quantitative reverse transcription PCR (qRT-PCR) analysis

Total RNA from three biological replicates was extracted using the Eastep Super Total RNA Extraction Kit (Promega, Madison, WI, USA). After confirming that RNA was of high quality, it was reverse-transcribed into cDNA using GoScript Reverse Transcription Mix (Promega). qRT-PCR was conducted with the SYBR Green system (Bio-Rad, Hercules, CA, USA). The primers used for qRT-PCR analysis were designed based on the National Center for Biotechnology Information (NCBI) database and are listed in Table S12. The maize *ZmUBQ1* (*Zm00001d015327*) gene was used as the internal control for normalization of gene expression levels.

### Measurement of fresh and dry weights

The maize B73 and Mo17 inbred lines, and two F_1_ hybrids (BM and MB) were grown in darkness or under far-red (2.5 μmol m^−2^ s^−1^), red (30.0 μmol m^−2^ s^−1^), blue (6.0 μmol m^−2^ s^−1^), or white (30.0 μmol m^−2^ s^−1^) light at 28 °C for 7 days. The aboveground portions of seedlings of all four genotypes were collected for measurement of fresh weight (*n* = 6–11). Dry weight was obtained after desiccation for 4 days at 65 °C. Mid-parent heterosis (MPH) for fresh and dry weights was calculated using the following formula: MPH = 100% × (F_1_ − A)/A, where F_1_ and A are the average values from three biological replicates for the hybrids and parents, respectively.

## Results

### Light quality affects gene expression in maize hybrids and their parents

**Fig. 1:**
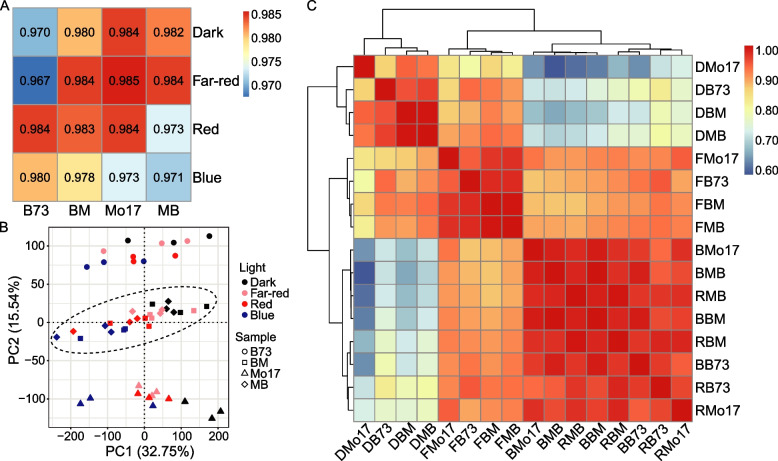
Global characterization of gene expression patterns in maize inbred parents and reciprocal hybrids. **A** Average correlation coefficients based on gene expression values among three biological replicates of F_1_ hybrids and two parents. **B** Principal component analysis (PCA) of maize transcriptomes for four genotypes grown under various light conditions. The ellipse represents hybrid samples. **C** Clustering analysis of transcript abundance profiles under various light conditions. Biological replicates are shown as individuals. BM and MB represent the F_1_ hybrids B73 × Mo17 and Mo17 × B73, respectively. DB73, DMo17, DBM, and DMB represent B73, Mo17, F_1_ hybrid BM, and F_1_ hybrid MB grown in darkness, respectively; FB73, FMo17, FBM, and FMB represent B73, Mo17, BM, and MB grown under far-red light condition, respectively; BB73, BMo17, BBM, and BMB represent B73, Mo17, BM, and MB grown under blue light condition, respectively; and RB73, RMo17, RBM, and RMB represent B73, Mo17, BM, and MB grown under red light condition, respectively

To investigate the effect of different light qualities on heterosis, and based on the research results of Lorrain et al. [43], gene expression levels of three biological replicates were examined in seedlings of the maize inbred lines B73 and Mo17, their F_1_ hybrid BM, and the reciprocal hybrid MB. Plants were grown in darkness for 6 days, followed by transfer to far-red, red, or blue light conditions, or were kept in darkness for 24 h (Fig. S1). The Q30 base percentage of raw reads was > 95%, and > 90% of the 2,329.4 Mb of clean reads (Table S1) was successfully mapped to the B73 RefGen_v4 genome (https://www.maizegdb.org/assembly/) [29]. The FPKM values of all identified genes were used to perform the correlation analysis. The average R^2^ values of the three biological replicates ranged from 0.967 to 0.985 (Fig. 1A), indicating that the transcriptome data generated in this study were highly reproducible.

PCA was performed for the identified genes in all four genotypes under various light conditions. Notably, gene expression levels in both hybrids clustered into a single group that was clearly separated from the two individual inbred lines based on principal component (PC) 2 (Fig. 1B). This clustering indicated significant changes in the overall gene expression landscape caused by hybridization. PC1 explained 32.75% of the total variance, with samples showing a moderate clustering tendency according to the light conditions (Fig. 1B). Through subsequent clustering analysis, we identified three distinct groups, with the four genotypes grown in darkness or under far-red light clustering into one group and the other two groups comprising a mixture of genotypes grown under red and blue light conditions. This clustering pattern suggested that red and blue light lead to similar expression patterns across inbred lines and hybrids (Fig. 1C).

### Differential gene expression patterns in maize hybrids grown under various light conditions

**Fig. 2:**
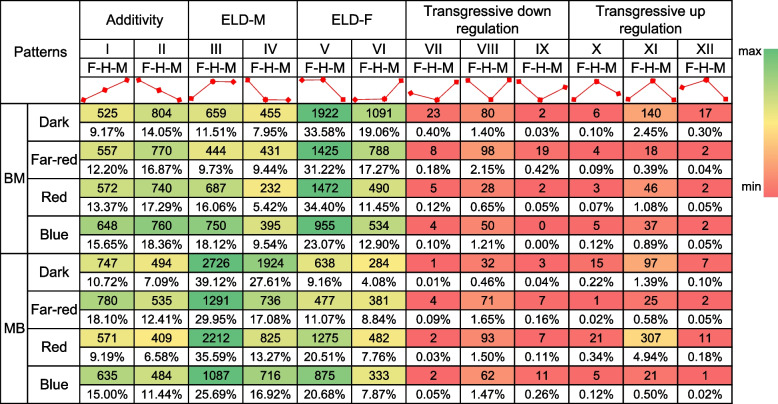
Number of parental expression level dominance (ELD) genes in maize hybrids. F–H-M, female parent-hybrid-male parent; ELD-F, genes with expression levels similar to the female parent in the F_1_ hybrid; ELD-M, genes with expression levels similar to the male parent in the F_1_ hybrid; BM and MB represent F_1_ hybrids B73 × Mo17 and Mo17 × B73, respectively. Fold change (FC) ≥ 1.5 or ≤  − 1.5 and false discovery rate (FDR) < 0.3

To explore how expressed genes responded to different light conditions, DEGs were classified into 12 female-hybrid-male (F–H-M) expression patterns (Fig. 2) according to the methods of Shen et al. [38]. DEGs with similar expression levels to those in the male and female parents were further designated as expression level dominance (ELD)-M genes (types III and IV) and ELD-F genes (types V and VI), respectively. Among these genes, additive (types I and II), complete-incomplete dominant (types III, IV, V, and VI) and overdominant (types VII, VIII, IX, X, XI, and XII) genes of both BM and MB accounted for 15.77–34.01%, 63.62–79.97%, and 2.01–7.10% of all DEGs, respectively. Moreover, type V genes in BM and type III genes in MB with similar expression levels to the B73 parent were most common (Fig. 2; Table S2). These observations suggested that non-additive genes in both hybrids contributed to maize heterosis under various light conditions, which is consistent with previous studies [15, 44, 45].

**Fig. 3:**
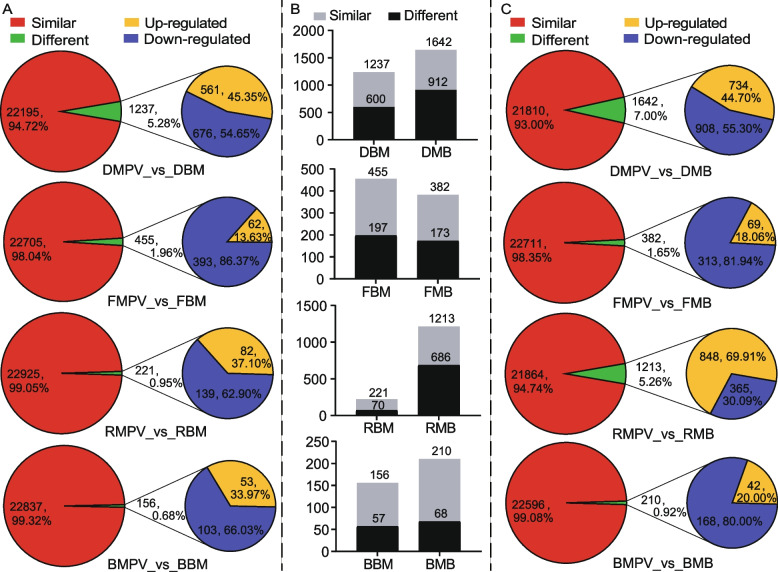
Overview of differentially expressed genes (DEGs) in F_1_ hybrids compared to mid-parent values (MPVs). **A**, **C** Number of genes differentially expressed in the F_1_ hybrids compared to the expected MPV (FC ≥ 1.5 or ≤  − 1.5 and FDR < 0.3) under various light conditions. **B** F_1_-MPV DEGs were frequently associated with DEGs between the two parents (represented by gray bars). DMPV, FMPV, RMPV, and BMPV represent MPV for plants grown in darkness and under far-red, red, and blue light conditions, respectively. DMB, FMB, RMB, and BMB represent the F_1_ hybrid MB grown in darkness and under far-red, red, and blue light conditions, respectively. DBM, FBM, RBM, and BBM represent the F_1_ hybrid BM grown in darkness and under far-red, red, and blue light conditions, respectively

F_1_-MPV DEGs are another potential factor affecting the establishment of heterosis [38]. F_1_-MPV DEGs (FC ≥ 1.5 or ≤  − 1.5 and FDR < 0.3) in the two hybrids grown in darkness or under far-red, red, or blue light conditions were analyzed. A small number of all identified genes (156 − 1642 or 0.68 − 7.00%) were F_1_-MPV DEGs and the proportions of F_1_-MPV DEGs in BM (5.28%, 0.95%, and 0.68%) were generally lower than those in MB (7.00%, 5.26%, and 0.92%) when grown in darkness and under red or blue light conditions, respectively **(**Fig. 3A, C; Fig. S2A-D; Table S3). Moreover, the number of downregulated genes was greater than that of upregulated genes in both BM and MB grown in darkness and under far-red and blue light conditions (1.20- and 1.24-fold, 6.34- and 4.54-fold, and 1.94- and 4.00-fold, respectively). Under red light, the number of downregulated genes was 1.70 times that of upregulated genes in BM. In contrast, the number of upregulated genes was 2.32 times that of downregulated genes in MB (Fig. 3A, C; Table S3, χ^2^ test, *P* < 0.01 or 0.05). Interestingly, DEGs between the two parents accounted for 43.45–68.33% of F_1_-MPV DEGs under different light conditions (Fig. 3B; Table S4), indicating that DEGs between the two parents may play an important role in the establishment of maize heterosis under various light conditions.

### Distinct regulatory networks of DEGs responded to various light conditions

To our knowledge, F_1_-MPV DEGs under various light conditions has not yet been systematically analyzed. In the present study, UpSet plots were used to reveal the distribution of F_1_-MPV DEGs. Both hybrids had the most F_1_-MPV DEGs in darkness (2172), followed by red (1293), far-red (655), and blue light (290). Comparing hybrids across the different light conditions, the comparisons of darkness–red and darkness–far red–red light possessed 235 and 73 common F_1_-MPV DEGs, respectively. Moreover, 78 F_1_-MPV DEGs were affected by all four light conditions (Fig. S2E). These data suggest that some DEGs had expression patterns specific to certain light conditions, while others were more universal.

**Fig. 4:**
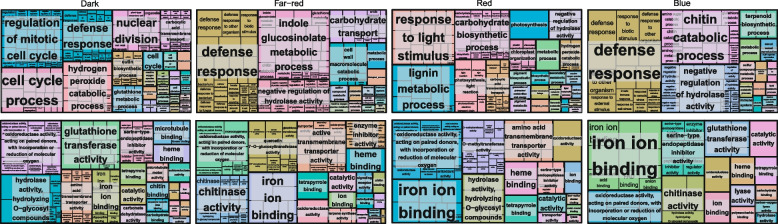
Gene Ontology (GO) term enrichment analysis of F_1_-MPV DEGs. Biological process and molecular function GO terms visualized using the “TreeMap” view of REVIGO, are displayed at the top and bottom, respectively. Each rectangle shows a single cluster representative. The representatives are shown in with different colors. The size of the rectangles reflects *P*

Next, GO enrichment analysis was performed for F_1_-MPV DEGs. A total of 328, 242, 234, and 127 significant GO terms (*P* < 0.05) were identified in darkness and under far-red, red, and blue light conditions, respectively (Table S5). These GO terms were further visualized using the “TreeMap” view of REVIGO (Fig. 4; Fig. S3). In darkness, F_1_-MPV DEGs were mainly related to the biological processes “mitotic cell cycle” (72 out of 1537, 4.68%), “defense response” (168 out of 1537, 10.93%), “nuclear division” (93 out of 1537, 6.05%), and “cell cycle” (84 out of 1537, 5.47%). F_1_-MPV DEGs produced under far-red light condition were associated with the biological processes “defense response” (75 out of 547, 13.71%), “indole glucosinolate metabolic process” (44 out of 547, 8.04%), and “carbohydrate transport” (41 out of 547, 7.5%). However, DEGs were significantly enriched for the biological processes “response to light stimulus” (101 out of 1010, 10.00%), “carbohydrate biosynthetic process” (89 out of 1010, 8.51%), “photosynthesis” (54 out of 1010, 5.35%), and “lignin metabolic process” (26 out of 1010, 2.57%) under red light condition. F_1_-MPV DEGs were mainly involved in the biological processes “terpenoid biosynthesis” (14 out of 228, 6.14%), “defense response” (41 out of 228, 17.98%), “chitin catabolic process” (32 out of 228, 14.04%), and “negative regulation of hydrolase activity” (16 out of 228, 7.02%) under blue light condition, (Fig. 4; Fig. S3A, B; Table S5).

In the molecular function GO category, F_1_-MPV DEGs were significantly enriched in “oxidoreductase activity” and “iron ion binding”. Of these DEGs, 7.48% (115 out of 1537), 5.85% (32 out of 547), 6.04% (61 out of 1010), and 3.51% (8 out of 228) were specifically enriched in “glutathione transferase activity”, “active transmembrane transporter activity”, “heme binding”, and “serine-type endopeptidase inhibitor activity” in darkness and under far-red, red, and blue light conditions, respectively (Fig. 4; Fig. S3C, D; Table S5). In darkness, DEGs might respond to light starvation by altering the activity of glutathione transferase (GST), to maintain plant growth and development through regulation of the cell cycle, nuclear division, and oxidoreductase activity. DEGs might influence carbohydrate transport under far-red light conditions, and indole glucosinolate metabolic processes and terpenoid biosynthesis under blue light conditions. In addition, when plants are stimulated with red light, DEGs might affect chlorophyll synthesis and metabolism, and photosynthetic efficiency, by regulating iron ion and heme binding. These results indicate that heterosis-related DEGs were affected by light conditions, likely through changes in regulatory networks.

**Fig. 5:**
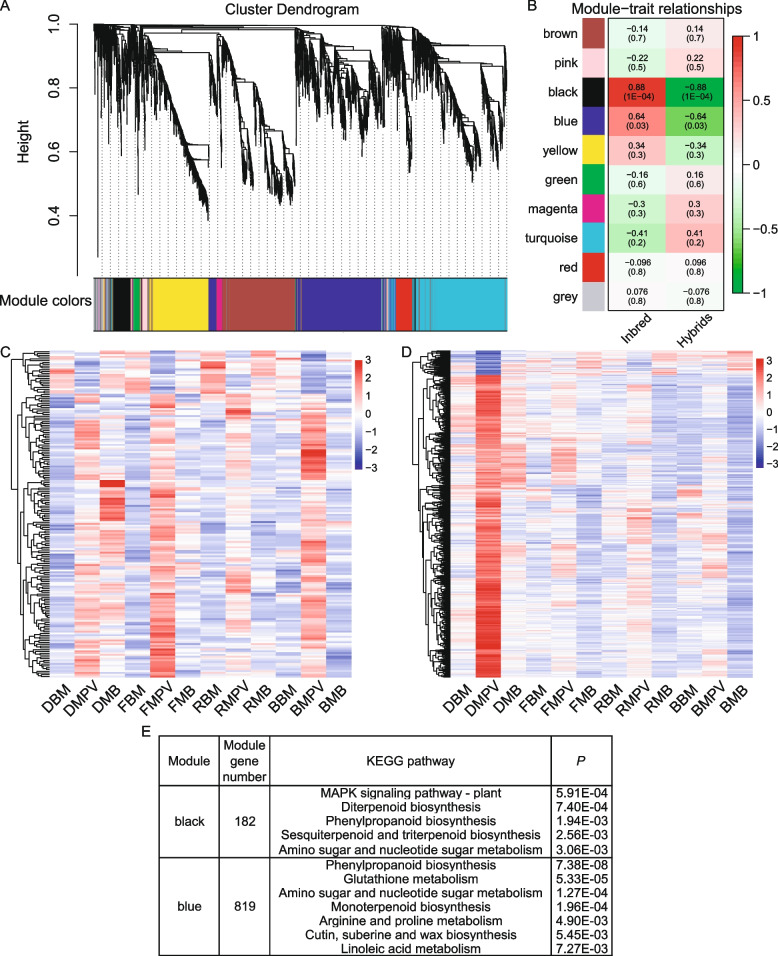
Weighted gene coexpression network analysis (WGCNA) of F_1_-MPV DEGs. **A**-**B** Co-expression networks were generated for 3366 F_1_-MPV DEGs from the transcriptome dataset. **C**-**D** Clustering analysis of co-expressed genes in black (**C**) and blue (**D**) modules. **E** Table showing the numbers of genes and Kyoto Encyclopedia of Genes and Genomes (KEGG) pathways within each module. The gray module represents unclustered genes. DBM and DMB represent the F_1_ hybrids BM and MB grown in darkness, respectively. FBM and FMB represent the F_1_ hybrids BM and MB grown under far-red light, respectively. RBM and RMB represent the F_1_ hybrids BM and MB grown under red light, respectively. BBM and BMB represent the F_1_ hybrids BM and MB grown under blue light, respectively. DMPV, FMPV, RMPV, and BMPV represent the expected MPVs of plants grown in darkness and under far-red, red, and blue light conditions, respectively

To reveal the coregulatory network among DEGs under various light conditions, WGCNA [39] was performed on 3366 F_1_-MPV DEGs, resulting in nine distinct modules and one gray module of unclustered genes (Fig. 5A). As shown in Fig. 5B, genes in both the black (r = 0.88, *P* = 1.0E − 04) and blue modules (*r* = 0.64, *P* = 0.03) were significantly positively correlated with gene expression in inbred lines, and negatively correlated with gene expression in hybrids (*r* =  − 0.88 or − 0.64, and *P* = 1.0E − 04 or 0.03, respectively) (Fig. 5B). F_1_-MPV DEGs of both the black and blue modules were subjected to cluster analysis. Clustering showed that gene expression differences between F_1_ and MPV in the black module were greater than those in the blue module (Fig. 5C, D), consistent with the results of module–trait correlation analysis (Fig. 5B). KEGG analysis indicated that F_1_-MPV DEGs in the black and blue modules were significantly enriched in “terpenoid biosynthesis”, “amino sugar and nucleotide sugar metabolism”, and “phenylpropanoid biosynthesis” (Fig. 5E; Table S6). Taken together, these results indicate that light-responsive, heterosis-related genes are likely involved in a wide array of biological processes, including defense, photosynthetic efficiency, and photosynthetic metabolism.

### Interaction effects of genotype and light on metabolite accumulation in maize hybrids

To assess the interactive effect of DEGs and light conditions on overall metabolism, non-targeted metabolomes of B73, Mo17, BM, and MB seedlings (grown in darkness for 6 days and subsequently transferred to darkness, far-red, red, or blue light for 24 h) were examined using LC–MS. The R^2^ values of the six biological replicates ranged from 0.979 to 0.995 (Fig. S4A), indicating that the overall quality of the metabolomic data was high. Similar to the gene expression PCA, the metabolome PCA showed a clear separation of hybrids from inbred lines along PC2 (14.06%). Light conditions were clearly separated along PC1 in all four genotypes (Fig. S4B), consistent with the results of transcriptome analysis. However, clustering analysis of the four genotypes under various light conditions resulted in mixed clusters (Fig. S4C). Two-way analysis of variance confirmed that both genotype and light affected the metabolomes of all four genotypes (*P* < 0.05). Out of the 2497 identified metabolites, 2209 (88.47%) and 2245 (89.91%) were significantly altered based on genotype and light condition, respectively, and 2122 (84.96%) were affected by both genotype and light condition (Fig. S4D; Table S7). Therefore, the interaction between genotype and light condition may explain the mixed clustering of metabolite profiling data.

**Fig. 6:**
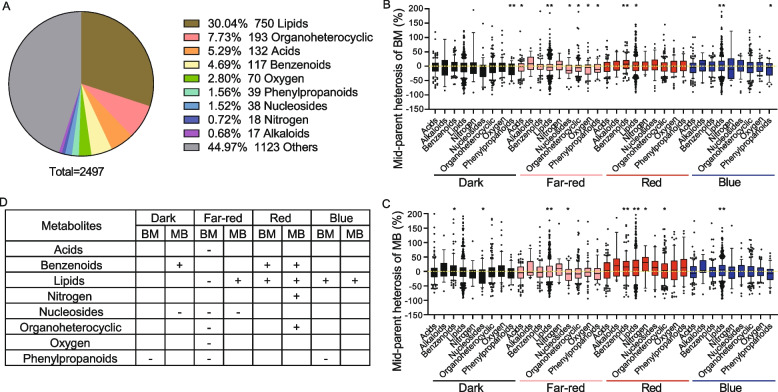
Light regulation of metabolic mid-parent heterosis (MPH) in maize. **A** Pie chart showing the relative composition of metabolites under various light conditions. **B**-**C** Boxplots showing the distribution of MPH for each known metabolite under various light conditions. MPH = 100% × (F_1_ − A)/A, where F_1_ is the metabolite accumulation in hybrids, and A is the average parental accumulation of metabolites. **D** Relationship between metabolites and maize MPH. BM and MB represent the F_1_ hybrids B73 × Mo17 and Mo17 × B73, respectively. “ − ” represents negative MPH; “ + ” represents positive MPH; ** represents a significant difference at *P* < 0.01; * represents a significant difference at *P* < 0.05 (one-sample *t*-test)

The mixed clustering of all four genotypes grown under different light conditions enabled the investigation of light effect on maize metabolome. The 2497 detected metabolites were divided into 10 categories, in which 1123 metabolites (44.97%) were not annotated and 30.04% were lipids, followed by metabolites classified as organoheterocyclic (7.73%), acids (5.29%), benzenoids (4.69%), oxygen (2.80%), phenylpropanoids (1.56%), and nucleosides (1.52%) (Fig. 6A). To quantitatively assess DAMs in response to different light conditions, the percent MPH for each annotated metabolite was calculated. The responses of nucleoside and phenylpropanoid metabolites to darkness and far-red light conditions was negatively regulated by maize MPH, the responses of lipid and benzenoid compounds to red light were positively regulated by maize MPH, and the response of lipids to blue light was positively regulated by maize MPH (Fig. 6B-D).

To further explore whether the classification of DAMs was similar to that of DEGs under different light conditions, 624 and 618 DAMs of BM and MB were divided into 12 categories, in which non-additive metabolites (types III, IV, V, VI, VII, VIII, IX, X, XI, and XII) accounted for 88.72–96.22% of all DAMs (Fig. S5A; Table S8), similar to the DEG analysis. In addition, 599 non-additive metabolites deviated from MPVs (Fig. S5B). Among those metabolites, the number of upregulated (higher than MPV) metabolites was very similar to the number of downregulated (lower than MPV) metabolites in hybrids grown in darkness and under blue light condition, as well as in BM grown under red light condition. Under far-red light condition, the number of downregulated metabolites was significantly higher than that of upregulated metabolites in both BM and MB (2.54- and 2.03-fold, respectively). In contrast, the number of upregulated metabolites in MB under red light condition was 2.38-fold higher than the number of downregulated metabolites (Fig. S5B; Table S9). These results were not consistent with those of F_1_-MPV DEGs due to the regulation of metabolites by multiple genes or signal transduction pathways. Moreover, under darkness, far-red, red, and blue light conditions, 100% (84/84), 98.99% (98/99), 100% (44/44), and 98.59% (70/71) of non-additive metabolites were shared by both BM and MB, respectively, and most overlapping non-additive metabolites showed similar expression changes (84/84, 100%; 98/99, 98.99%; 44/44, 100%; and 70/71, 98.59%; respectively) (Fig. S5C). Taken together, these findings suggest that non-additive metabolites play an important role in maize heterosis under various light conditions, with patterns similar to gene expression patterns.

### Integration of metabolite and gene expression data for light-specific pathways

**Fig. 7:**
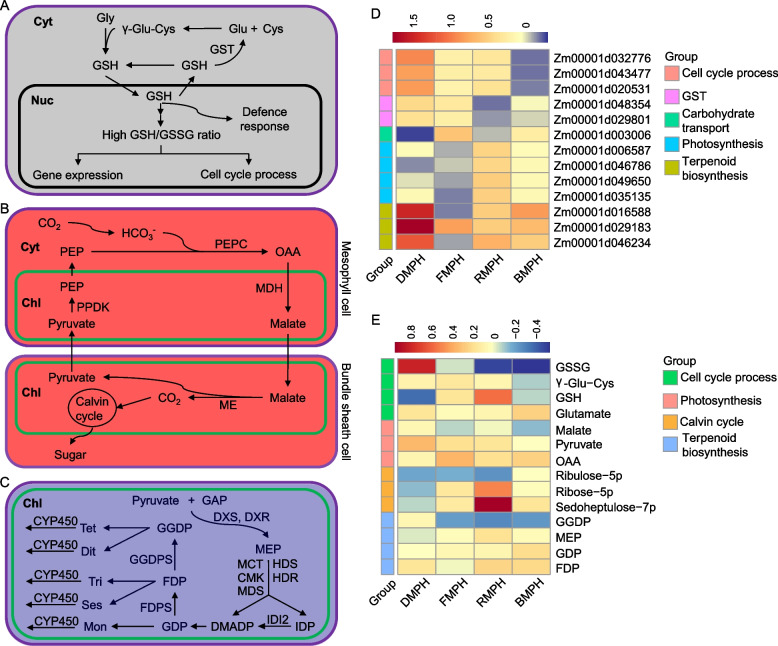
Specific genes and metabolites involved in maize heterosis establishment under various light conditions. **A** Proposed pathway describes the action of glutathione transferases (GSTs) in darkness. The pathway indicates that glutathione (GSH) is synthesized in two steps: first, γ-glutamyl-cysteine (γ-Glu-Cys) is formed from glutamate (Glu) and cysteine (Cys), followed by the addition of glycine (Gly) by glutathione synthetase. GSH is generally found in the nucleus (Nuc) and cytoplasm (Cyt), and the GSH/GSSG (glutathione disulfide) ratio in the nucleus influences gene expression, the cell cycle, and defense responses. Furthermore, GST can catalyze GSH to regenerate Glu. **B** The pathway of photosynthesis under red light condition proposed by Li et al. [51] with minor modifications. First, CO_2_ enters the mesophyll cytoplasm (Cyt) and is converted to bicarbonate (HCO_3_^−^). HCO_3_^−^ and phosphoenolpyruvate (PEP) produce oxaloacetate (OAA) under the action of phosphoenolpyruvate carboxylase (PEPC). OAA is converted to malate by malate dehydrogenase (MDH) in the mesophyll chloroplast (Chl), and this malate diffuses into the chloroplasts of bundle sheath cells. Malate is decarboxylated by malic enzyme (ME) to produce pyruvate and CO_2_. Finally, CO_2_ enters the Calvin cycle and is fixed, thus producing sugar. In addition, pyruvate is recruited to mesophyll cells and converted to PEP by pyruvate orthophosphate dikinase (PPDK). **C** Proposed biosynthesis of terpenoids via the 2-*C*-methylerythritol 4-phosphate (MEP) pathway under blue light conditions. The pathway is simplified from Nagegowda et al. [52]. Terpenoids are synthesized from isopentenyl diphosphate (IDP) and its allylic isomer dimethylallyl diphosphate (DMADP), the products of the MEP pathway in plastids. The MEP pathway starts with pyruvate and glyceraldehyde-3-phosphate (GAP), which undergo a series of enzymatic reactions conducted by 1-deoxy-D-xylulose-5-phosphate synthase (DXS), 1-deoxy-D-xylulose-5-phosphate reductoisomerase (DXR), and 2-*C*-methyl-D-erythritol 4-phosphate cytidylyltransferase (MCT) to produce isopentenyl diphosphate (IDP) and dimethylallyl diphosphate (DMADP). The resulting terpenoids are then modified by cytochrome P450 monooxygenase (CYP450). (D) Mid-parent heterosis (MPH) of candidate genes under various light conditions. (E) MPH of candidate metabolites under various light conditions. Abbreviations: CMK, 4-(cytidine 5′-diphospho)-2-*C*-methyl-D-erythritol kinase; MDS, 2-*C*-methyl-D-erythritol 2,4-cyclodiphosphate synthase; HDS/HDR, 4-hydroxy-3-methylbut-2-enyl diphosphate synthase/reductase; IDI2, isopentenyl diphosphate isomerase; GDP, geranyl diphosphate; FDPS, farnesyl diphosphate synthase; GGDPS, geranylgeranyl diphosphate synthase; Mon, monoterpenes (C10); Ses, sesquiterpenes (C15); Tri, triterpenes (C30); Dit, diterpenes (C20); Tet, tetraterpenes (C40); CYP450, cytochrome P450 monooxygenase. DMPH, FMPH, RMPH, and BMPH represent MPH of plants grown in darkness and far-red, red, and blue light conditions, respectively

F_1_-MPV DEGs associated with darkness were mainly enriched in “cell cycle” and “glutathione transferase activity” (Fig. 4; Fig. S3). After combining DEGs and DAMs, the levels of glutamate (Glu) substrate and γ-glutamyl-cysteine (γ-Glu-Cys) intermediate in glutathione (GSH) synthesis were higher in hybrids compared to the MPV, while the expression levels of *GST* (*GST9*/*Zm00001d048354* and *GST14*/*Zm00001d029801*) catalyzing GSH to produce Glu, and the accumulation of glutathione disulfide (GSSG) produced by the reaction of GSH with oxidants also increased and GSH content decreased. In addition, the expression levels of cellulose synthase genes (*ZmCesA10*/*Zm00001d032776*, *ZmCesA11*/*Zm00001d043477*, and *ZmCesA12*/*Zm00001d020531*) (Fig. 7A, D; Table S10, S11) involved in the cell cycle [46] increased in hybrids compared to MPV. The GSH/GSSG ratio plays key roles in the maintenance of cellular redox homeostasis and tolerance of biotic and abiotic stresses [47, 48], and genes related to the cell cycle are associated with biomass heterosis [49, 50]. Thus, maize hybrids reduced the GSH/GSSG ratio by regulating the expression level of *GST* and the oxidation of GSH, and then affected gene expression, cell cycle progress, and defense responses. All of these interactions were further impacted by the action of DEGs in darkness.

Photosynthesis-related genes have been shown to be associated with biomass heterosis [49–51]. When plants were stimulated with red light, F_1_-MPV DEGs were mainly enriched in carbohydrate biosynthesis and photosynthesis (Fig. 4; Fig. S3). Accordingly, F_1_-MPV DAMs in the photosynthesis pathway under red light condition were compared, and sedoheptulose-7p and ribose-5 in the Calvin cycle showed positive MPH. This finding is consistent with the MPH of oxaloacetate and pyruvate metabolites involved in photosynthesis (Fig. 7B, E; Table S10). Intriguingly, F_1_-MPV DEGs involved in photosynthesis also showed positive MPH, including genes that encode chlorophyll-binding proteins (*Zm00001d006587* and *Zm00001d046786*) and photosystem II core complex proteins (*Zm00001d049650* and *Zm00001d035135*) (Fig. 7D; Table S11). These findings suggest that reciprocal hybrids had higher photosynthetic efficiency than the two inbred parents, which might partly explain the biomass heterosis observed in maize hybrids grown under red light.

Under far-red light, F_1_-MPV DEGs were specifically enriched in carbohydrate transport (Fig. 4; Fig. S3), and the *PIP2; 5* (*Zm00001d003006*) gene displayed positive MPH in reciprocal hybrids (Fig. 7D; Table S11). However, no F_1_-MPV DAMs were involved in carbohydrate transport. Therefore, under far-red light, hybrids might increase the *PIP2;5* expression level to enhance carbohydrate transport and heterosis.

When plants were moved from darkness to blue light, F_1_-MPV DEGs were significantly enriched in terpenoid biosynthesis and defense responses (Fig. 4; Fig. S3). Among these genes, *Zm00001d016588* (glycerophosphodiester phosphodiesterase), *Zm00001d029183* (cytochrome P450 oxygenase, CYP450), and *Zm00001d046234* (myo-inositol oxygenase) showed positive MPH (Fig. 7D; Table S11). Similarly, DAMs of 2-*C*-methylerythritol 4-phosphate (MEP), geranyl diphosphate (GDP), and farnesyl diphosphate (FDP) associated with the synthesis of triterpenes (Tri), sesquiterpenes (Ses), and monoterpenes (Mon) showed positive MPH. Nevertheless, geranylgeranyl diphosphate (GGDP) participating in the synthesis of diterpenes (Dit) and tetraterpenes (Tet) showed negative MPH (Fig. 7C, E; Table S10). Terpenoids play important roles in plant stress responses and defense mechanisms [52]. Taken together, these results indicate that maize hybrids might show improved plant adaptability to blue light driven by an elevated concentration of terpenoids.

To verify the expression levels of light-specific DEGs, qRT-PCR analyses of were performed on 13 candidate genes involved in cell cycle, photosynthesis, carbohydrate transport, and terpenoid biosynthesis (Table S12). Most qRT-PCR results were consistent with the RNA-seq data, with the exception of *Zm00001d029801* and *Zm00001d020531* in darkness, and *Zm00001d046786* under red light (Fig. S6). Overall, the qRT-PCR data demonstrated the reliability of RNA-seq quantifications.

### Phenylpropanoid biosynthesis is a common pathway under various light conditions

To explore the common DAMs in response to various light conditions, 599 F_1_-MPV DAMs were analyzed by WGCNA and divided into three distinct modules and one gray module of unclustered metabolites (Fig. S7A). According to correlation analysis, including of traits and expression modules, the brown module was significantly positively correlated with maize inbred lines (r = 0.93, *P* = 9.0E − 06) and negatively correlated with hybrids (*r* =  − 0.93, *P* = 9.0E − 06) (Fig. S7B). Further, cluster analysis was performed on the accumulation of metabolites in the brown module. These metabolites were clearly divided into two categories: F_1_ > MPV and MPV < F_1_ (Fig. S7C). Additionally, KEGG enrichment analysis of DAMs in the brown module revealed that these DAMs were enriched mainly in phenylpropanoid and flavonoid biosynthesis (Fig. S7D). Flavonoids are phenolics produced via the phenylpropanoid biosynthesis pathway, which are important for plant growth, development, and defense responses as anti-pathogenic, antioxidant, or UV-absorbing compounds, or as signaling molecules mediating plant–microbe interactions [53, 54]. Therefore, the phenylpropanoid–flavonoid biosynthesis might be a common metabolic pathway contributing to maize heterosis under different light conditions. The result was consistent with WGCNA of DEGs.

Based on the transcriptome data, phenylalanine ammonia-lyase (*PAL*), cinnamate 4–hydroxylase (*C4H*), and chalcone synthase (*CHS*) in the phenylpropanoid − flavonoid biosynthesis pathway had positive or negative MPH shared between two to three light conditions (Fig. S8A, B; Table S13). qRT-PCR analysis confirmed that the RNA-seq data were reliable (Fig. S8D). However, minor variations were observed in genes across the four light conditions. In addition, based on the metabolomic data obtained under different light conditions, almost all L-phenylalanine and cinnamoyl-CoA metabolites in phenylpropanoid biosynthesis showed positive MPH, while the intermediate metabolites delphinidin, ( +)-catechin, chlorogenic acid, pinocembrin, and tricetin in flavonoid biosynthesis showed consistently negative MPH (Fig. S8A, C; Table S14). Most of these changes would repress defense responses, indicating that the defenses of maize hybrids may be improved through other pathways and phenylpropanoid-flavonoid pathway will be advantageous to the heterosis for growth and development.

To further validate the accuracy of the expression data for the candidate genes obtained from RNA-seq analysis, 45 genes (Fig. S9A) involved in several pathways were selected for qRT-PCR analysis. When comparing the qRT-PCR expression and RNA-seq data, a large Pearson correlation coefficient (R^2^ = 0.9617) was obtained (Fig. S9B, C). These results confirmed the reliability of the RNA-seq data.

### Effects of light conditions on maize biomass heterosis

Heterosis is closely related to environmental conditions [55]. As maize phenotypes were not strongly affected by differences in light conditions over 24 h, the fresh and dry weights of maize inbred lines (B73 and Mo17), and their reciprocal F_1_ hybrids, grown in darkness, or under far-red, red, blue, or white light for 7 days, were measured to determine the effect of light conditions on biomass heterosis (Fig. S10A-E). Through quantification of biomass heterosis (Table S15), increased light intensity (far-red, blue, red, or white light) resulted in higher levels of MB biomass heterosis based on both fresh and dry weights, while MB hybrids grown in darkness had moderate biomass heterosis (62.01% and 55.13%, respectively). Interestingly, BM biomass heterosis decreased with increasing light intensity (darkness, far-red, blue, or red light), although it had relatively high fresh and dry weight biomass heterosis (42.11% and 45.09%, respectively) under white light. Overall, the hybrid heterosis of fresh weight was similar to that of dry weight under various light conditions, but the biomass heterosis of MB was significantly higher than that of BM (Fig. S10F). These results indicate that the heterosis of MB was significantly impacted by light conditions, which provides a basis for utilizing light conditions to enhance biomass heterosis.

## Discussion

### Light-specific and general regulatory networks among DEGs and DAMs under various light conditions

As a crucial environmental signal and driving force of photosynthesis, light has significant impacts on many aspects of plant growth and development, including heterosis. Increasing plant density causes plants to compete for light to support photosynthesis. At high planting density, “paternal-effect” DEGs of maize F_1_ plants were the main participants in plant hormone production and abiotic/biotic stress responses to adapt to environmental stress. “Maternal-effect” DEGs were mainly involved in the synthesis of energy storage materials, including the processes of photosynthesis, carbohydrate biosynthesis, and metabolism [25]. The interaction mechanism between light and heterosis has been poorly studied. Here, the transcriptome and metabolome profiles of maize reciprocal hybrids and their parents under various light conditions were analyzed. The results enabled the identification of condition-specific and general interaction networks.

Light-specific and general regulatory networks among DEGs, DAMs, and environmental factors were revealed (Fig. 7; Fig. S8A**)**. Specifically, GSTs, carbohydrate transport, photosynthesis, and terpenoid biosynthesis involving defense, photosynthetic efficiency, and photosynthetic metabolism pathways were correlated with hybrid vigor under dark, far-red, red, and blue light conditions, respectively (Fig. 7). The phenylpropanoid–flavonoid biosynthesis pathway in hybrids was affected under all light conditions, making it the most consistently altered biological process associated with heterosis (**F**ig. S8A). Therefore, we speculate that under different light conditions, flavonoids produced via phenylpropanoid biosynthesis pathway will affect the heterosis of growth and development.

### Non-additivity of genes and metabolites contributes to maize heterosis under various light conditions

The expression patterns of DEGs revealed that most DEGs had non-additive expression (Fig. 2), in both specific and general pathways (Table S2). These results are consistent with previous research that non-additive genetic effects are major contributors to heterosis [56]. However, F_1_-MPV DEGs accounted for a small fraction of all identified genes under different light conditions (Fig. 3). This small number of non-additive F_1_ genes is similar to findings previously reported for *Brassica napus* [38]. Although a relatively small number of non-additive genes may have an outsized impact on heterosis, heterosis generally results from a large number of expression changes with small individual effects.

Notably, a small number of metabolites had non-additive values in both reciprocal hybrids (5.09–9.09% in MB and 4.77–8.01% in BM) (Fig. S5C), in sharp contrast to the large fractions reported previously [51, 57]. The high MPH of metabolites identified in this study (Fig. 6B, C) differs significantly from the mild MPH reported in previous studies [51, 58], although some researchers have reported effects of a similar magnitude [59, 60]. The extremely variable level of metabolite MPH among different experiments may result from differences in sampling time, species, tissues, or data analysis methods. In addition, the overlaps of non-additive genes (10.75–32.55%, Fig. S2A-D) and metabolites (14.77–36.04%, Fig. S5C) between reciprocal hybrids were relatively small in this study. Similar low-overlap results have been reported for gene expression data obtained from endosperm tissue [59] and metabolomics data from seedlings [51]. This tendency may be driven by the parent-of-origin effect, in which hybrid phenotypes can be strongly influenced by the selection of inbred lines for use as the male or female parent.

This study confirmed that non-additive genes and metabolites play important roles in specific and general interaction networks for maize genotypes and light conditions. However, more research is still needed to clarify the molecular mechanisms of heterosis–environment crosstalk. Furthermore, systematic characterization of changes in gene expression and metabolite levels will yield insights into heterosis that have important implications for crop breeding.

### Impacts of light-specific pathways on crop production

Heterosis has been used to dramatically increase maize yield for over a century [61]. However, even with recent technological advances, the genetic and molecular mechanisms underlying the phenomenon of heterosis remain elusive [28]. Heterosis represents a compound effect of multiple loci [62], and the expression of heterosis-related genes is a complex process influenced by genetic and epigenetic variations. This complexity is compounded by the impacts of environmental conditions on plant development [15, 18–21]. Previous studies have demonstrated the complexity of maize heterosis.

Light is one of the most important environmental factors affecting plant growth and development. In lettuce, far-red light results in sparse plant growth and reduced content of chlorophyll, carotenoids, and anthocyanins compared with white light [63, 64]. Far-red light promotes the accumulation of soluble sugar and nitrate, largely consistent with the changes in carbohydrate transport found in this study. Conversely, supplemental red and blue light increase the contents of chlorophyll, carotenoids, and anthocyanins, and increase the fresh weight of lettuce [63, 64]. Red light resulted in more compact and rapid growth, similar to the phenotype observed in the present study (Fig. 7B), while blue light led to plant dwarfing, possibly due to the activation of defense responses (Fig. 7C).

Suitable light is required to exploit the full yield potential of hybrids. The CCA1 protein regulates the plant circadian clock and promote photosynthesis, starch metabolism, and biomass heterosis under various light conditions [23, 65]. In the present study, both BM and MB hybrids showed positive MPH for shoot biomass under multiple light conditions, although the MB hybrid showed a stronger effect. The phenotypic differences of between reciprocal crosses are similar to the results of Ko et al. [65]. It may be that Mo17 grains are larger than B73 grains, which makes Mo17 store more nutrients and MB hybrids have stronger heterosis. Despite this similarity, the individual responses of these hybrids to light intensity differed. With increasing light intensity, the BM hybrid MPH based on fresh and dry weights decreased, while the MB hybrid showed the opposite trend (Fig. S10). This may be related to allele-specific expression. Different intensities trigger the expression of different superior alleles in BM and MB. Although genotype–light interaction networks and the effects of light conditions on maize seedling biomass heterosis were analyzed, the heterosis-related genes and metabolites driving the observed phenotypic differences remain to be clarified.

## Conclusions

This study is the first integration analysis of the transcriptome and metabolome of maize inbred lines B73 and Mo17, as well as their reciprocal hybrids in response to different light quality. Expression analysis revealed significant differences between hybrids and inbred lines. Most DEGs and DAMs showed non-additivity effects. Integration of DEGs and DAMs revealed that the biological processes of heterosis-related genes and metabolites were mainly focused on glutathione transfer, carbohydrate transport, photosynthesis and terpenoid biosynthesis under darkness, far-red, red and blue light conditions, respectively. In addition, the WGCNA results showed that hybrids exhibited advantages in the process of phenylpropanoid-flavonoid biosynthesis under all light conditions. Five genes and seven metabolites potentially playing roles in light-dependent heterotic effects were found. These genes and metabolites warrant further investigation to determine their impacts on biomass heterosis, as this could lead to improved hybrid breeding.

## Supplementary Information

**Additional file 1:** **Table S1.** Quality detection of RNA-seq data. **Table S2.** The classification of differentially expressed genes in both hybrids under various light conditions. **Table S3.** Differentially expressed genes in the comparisons of hybrids vs mid-parent value under various light conditions. **Table S4.** The comparisons of F_1_-MPV DEGs in both hybrids and DEGs between two parents. **Table S5.** Gene Ontology enrichment analyses for the F_1_-MPV DEGs under various light conditions. **Table S6.** Representative Kyoto Encyclopedia of Genes and Genomes (KEGG) pathway under various light conditions. **Table S7.** Details of two-way ANOVA for metabolites. **Table S8.** The patterns of differentially accumulated metabolites in BM and MB. **Table S9.** Differentially accumulated metabolites between hybrids and mid-parent values under various t light conditions. **Table S10.** The MPHs of specific metabolites under various light conditions. **Table S11.** Specific gene MPHs of RNA-seq data under various light conditions. **Table S12.** Primers for qRT-PCR. **Table S13.** Common gene MPHs of RNA-seq data under various light conditions. **Table S14.** The MPHs of common metabolites under various light conditions. **Table S15.** The biomass heterosis in maize seedlings.

**Additional file 2:** **Fig. S1.** Representative plants used for RNA-seq. **Fig. S2.** Analysis of differentially expressed genes (DEGs) under various light conditions. **Fig. S3.** GO enrichment categories of F_1_-MPV DEGs under various light conditions. **Fig. S4.** Patterns of light and genotypic effects on maize metabolomes. **Fig. S5.** Analysis of differentially accumulated metabolites in hybrids under various light conditions. **Fig. S6.** qRT-PCR confirmation of specific DEGs under various light conditions. **Fig. S7.** WGCNA analysis of F_1_-MPV differentially accumulated metabolites (DAMs). **Fig. S8.** Phenylpropanoid biosynthesis pathway under various light conditions. **Fig. S9.** MPH validation of 45 selected genes. **Fig. S10.** Biomass heterosis at the seedling stage under various light conditions.

## Data Availability

Transcriptome raw data in this study have been uploaded to the National Center for Biotechnology Information Sequence Read Archive (SRA) database (https://www.ncbi.nlm.nih.gov/sra; accession no. PRJNA780806) and the National Genomics Data Center Genome Sequence Archive (GSA) database (https://ngdc.cncb.ac.cn/gsa/; accession no. CRA006411).

## Abbreviations

GO: Gene ontology
KEGG: Kyoto encyclopedia of genes and genomes
WGCNA: Weighted gene co-expression network analysis
DEGs: Differentially expressed genes
DAMs: Differentially accumulated metabolites
RNA-seq: RNA sequencing
FPKM: Fragments per kilobase of transcript per million mapped reads
PCA: Principal component analysis
FC: Fold change
FDR: False discovery rate
LC–MS: Liquid chromatography–mass spectrometry
VIP: Variable importance in projection
MPH: Mid-parent heterosis
qRT-PCR: Real-time quantitative reverse transcription PCR
