# Supplementary material for: Combined transcriptome and metabolome analysis reveals the effects of light quality on maize hybrids
Source: BMC Plant Biol. 2023 Jan 18;23:41. doi: 10.1186/s12870-023-04059-4 (PMC9847186; doi:10.1186/s12870-023-04059-4)
Supplement: Supplementary file 2 — Additional file 2: Fig. S1. Representative plants used for RNA-seq. Fig. S2. Analysis of differentially expressed genes (DEGs) under various light conditions. Fig. S3. GO enrichment categories of F1-MPV DEGs under various light conditions. Fig. S4. Patterns of light and genotypic effects on maize metabolomes. Fig. S5. Analysis of differentially accumulated metabolites in hybrids under various light conditions. Fig. S6. qRT-PCR confirmation of specific DEGs under various light conditions. Fig. S7. WGCNA analysis of F1-MPV differentially accumulated metabolites (DAMs). Fig. S8. Phenylpropanoid biosynthesis pathway under various light conditions. Fig. S9. MPH validation of 45 selected genes. Fig. S10. Biomass heterosis at the seedling stage under various light conditions. [file 12870_2023_4059_MOESM2_ESM.docx]

**Combined transcriptome and metabolome analysis reveals the effects of light quality on maize hybrids**

Weimin Zhan^1#^, Guanghui Guo^2#^, Lianhua Cui^1^, Muhammad Abdul Rehman Rashid^3^, Liangliang Jiang^1^, Guanghua Sun^1^*, Jianping Yang^1^*, Yanpei Zhang^1^*

^1^ State Key Laboratory of Wheat and Maize Crop Science, Collaborative Innovation Center of Henan Grain Crops, College of Agronomy, Henan Agricultural University, Zhengzhou 450002, China

^2^ State Key Laboratory of Crop Stress Adaptation and Improvement, College of Agriculture, Henan University, Kaifeng 475004, China

^3^ Department of Bioinformatics and Biotechnology, Government College University Faisalabad, Faisalabad 38000, Pakistan

^#^ These authors contributed equally to this work

* Corresponding author: sgh126@126.com; jpyang@henau.edu.cn; zhangyanpei@henau.edu.cn

**Supplementary Information**

**Additional file 1: Table S1**: Quality detection of RNA-seq data.

**Additional file 2: Table S2**: The classification of differentially expressed genes in both hybrids under various light conditions.

**Additional file 3: Table S3**: Differentially expressed genes in the comparisons of hybrids vs mid-parent value under various light conditions.

**Additional file 4: Table S4**: The comparisons of F_1_-MPV DEGs in both hybrids and DEGs between two parents.

**Additional file 5: Table S5**: Gene Ontology enrichment analyses for the F_1_-MPV DEGs under various light conditions.

**Additional file 6: Table S6**: Representative Kyoto Encyclopedia of Genes and Genomes (KEGG) pathway under various light conditions.

**Additional file 7: Table S7**: Details of two-way ANOVA for metabolites.

**Additional file 8: Table S8**: The patterns of differentially accumulated metabolites in BM and MB.

**Additional file 9: Table S9**: Differentially accumulated metabolites between hybrids and mid-parent values under various t light conditions.

**Additional file 10: Table S10**: The MPHs of specific metabolites under various light conditions.

**Additional file 11: Table S11**: Specific gene MPHs of RNA-seq data under various light conditions.

**Additional file 12: Table S12**: Primers for qRT-PCR**.**

**Additional file 13: Table S13**: Common gene MPHs of RNA-seq data under various light conditions**.**

**Additional file 14: Table S14:** The MPHs of common metabolites under various light conditions.

**Additional file 15: Table S15**: The biomass heterosis in maize seedlings.

**Additional file 16: Fig. S1.** Representative plants used for RNA-seq.

**Additional file 17: Fig. S2.** Analysis of differentially expressed genes (DEGs) under various light conditions.

**Additional file 18: Fig. S3.** GO enrichment categories of F_1_-MPV DEGs under various light conditions.

**Additional file 19: Fig. S4.** Patterns of light and genotypic effects on maize metabolomes.

**Additional file 20: S5.** Analysis of differentially accumulated metabolites in hybrids under various light conditions.

**Additional file 21: Fig. S6.** qRT-PCR confirmation of specific DEGs under various light conditions.

**Additional file 22: Fig. S7.** WGCNA analysis of F_1_-MPV differentially accumulated metabolites (DAMs).

**Additional file 23: Fig. S8.** Phenylpropanoid biosynthesis pathway under various light conditions.

**Additional file 24: Fig. S9.** MPH validation of 45 selected genes.

**Additional file 25: Fig. S10.** Biomass heterosis at the seedling stage under various light conditions.

**
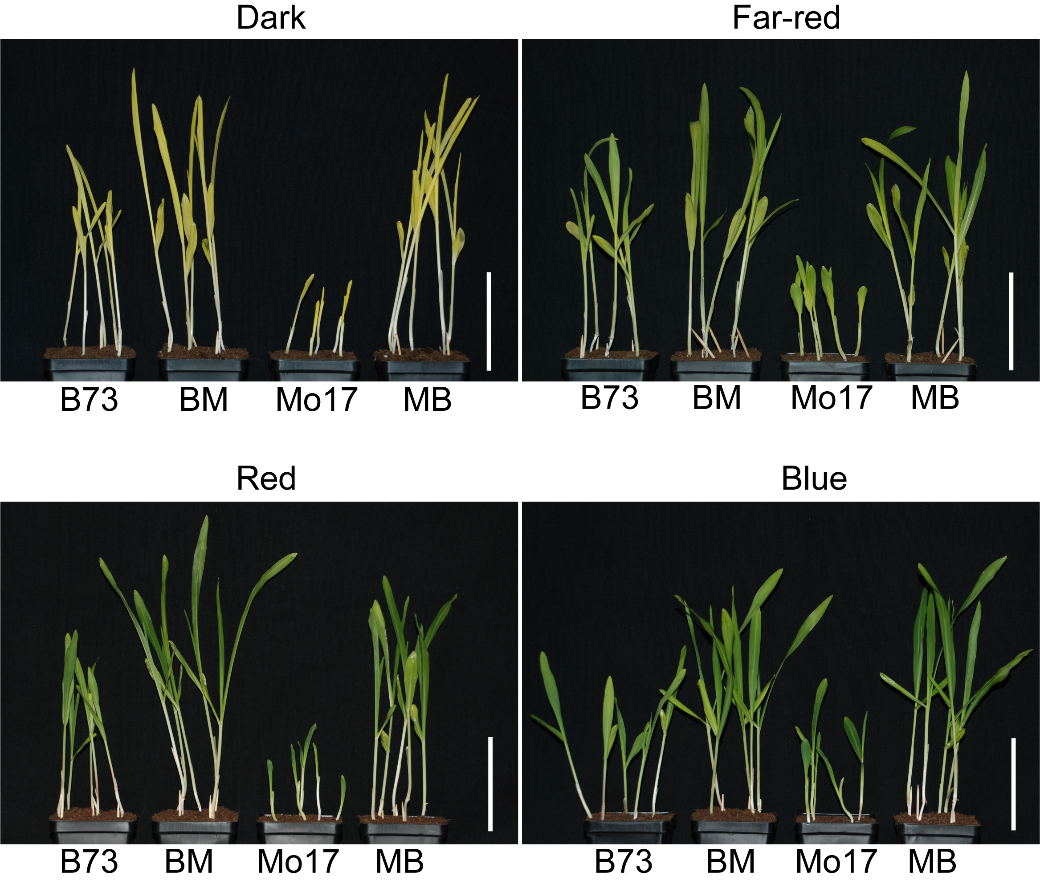
**

**Fig. S1.** Representative plants for RNA-seq. Maize seedlings were grown in darkness for 6 days and subsequently transferred to dark, far-red, red, or blue light conditions for 24 hours. BM and MB represent F_1_ hybrids B73×Mo17 and Mo17×B73, respectively. The scale bar represents 10 cm.

**
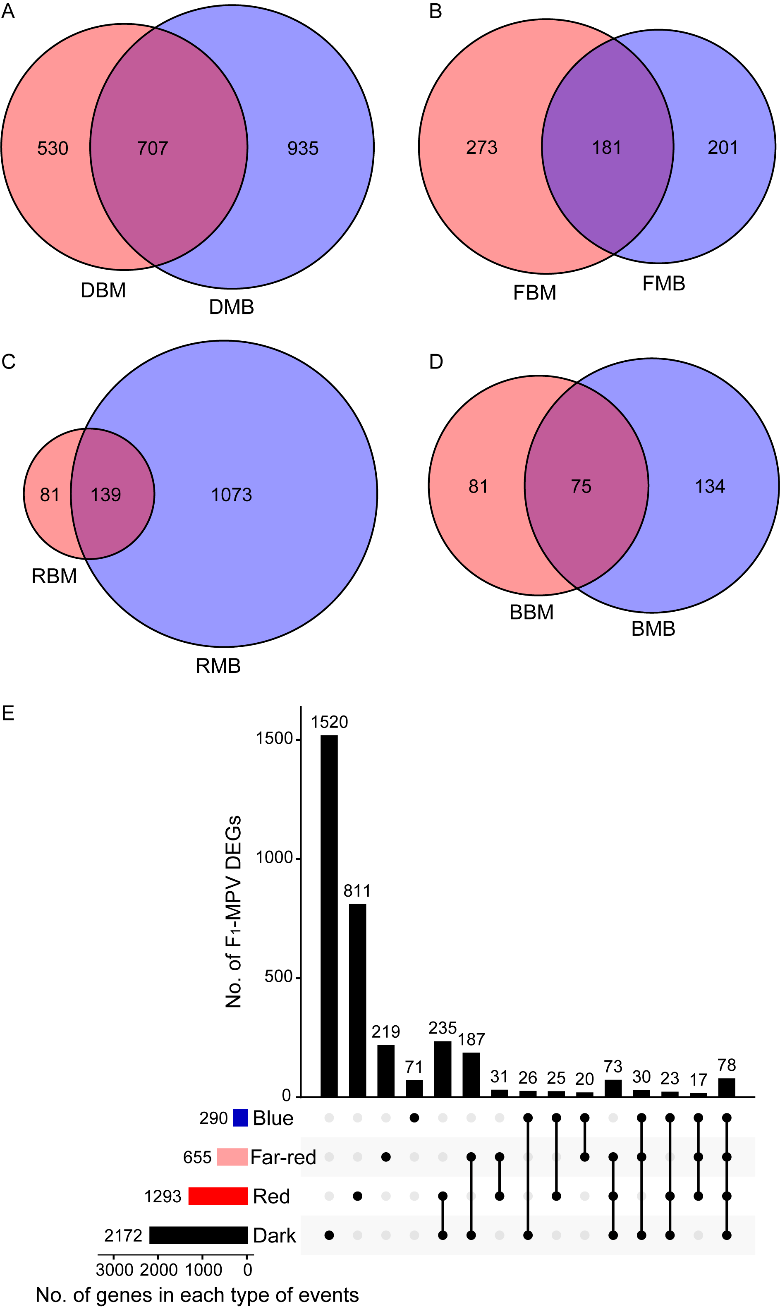
**

**Fig. S2.** Analysis of differentially expressed genes (DEGs) under various light conditions. (**A-D**) Overlap of non-additive genes between BM and MB under various light conditions. DMB, FMB, BMB, and RMB represent the F_1_ hybrid MB grown in darkness and under far-red, red, and blue light conditions, respectively; DBM, FBM, BBM, and RBM represent the F_1_ hybrid BM grown in darkness, and under far-red, red, or blue light conditions, respectively. (**E**) UpSet plot for F_1_-MPV DEGs under various light conditions. The upper histogram represents the number of F_1_-MPV DEGs in each group. The histogram at the bottom left represents the total number of F_1_-MPV DEGs in plants grown in darkness and under far-red, red, and blue light conditions, respectively. The dots and lines at the bottom right indicate subsets of F_1_-MPV DEGs.


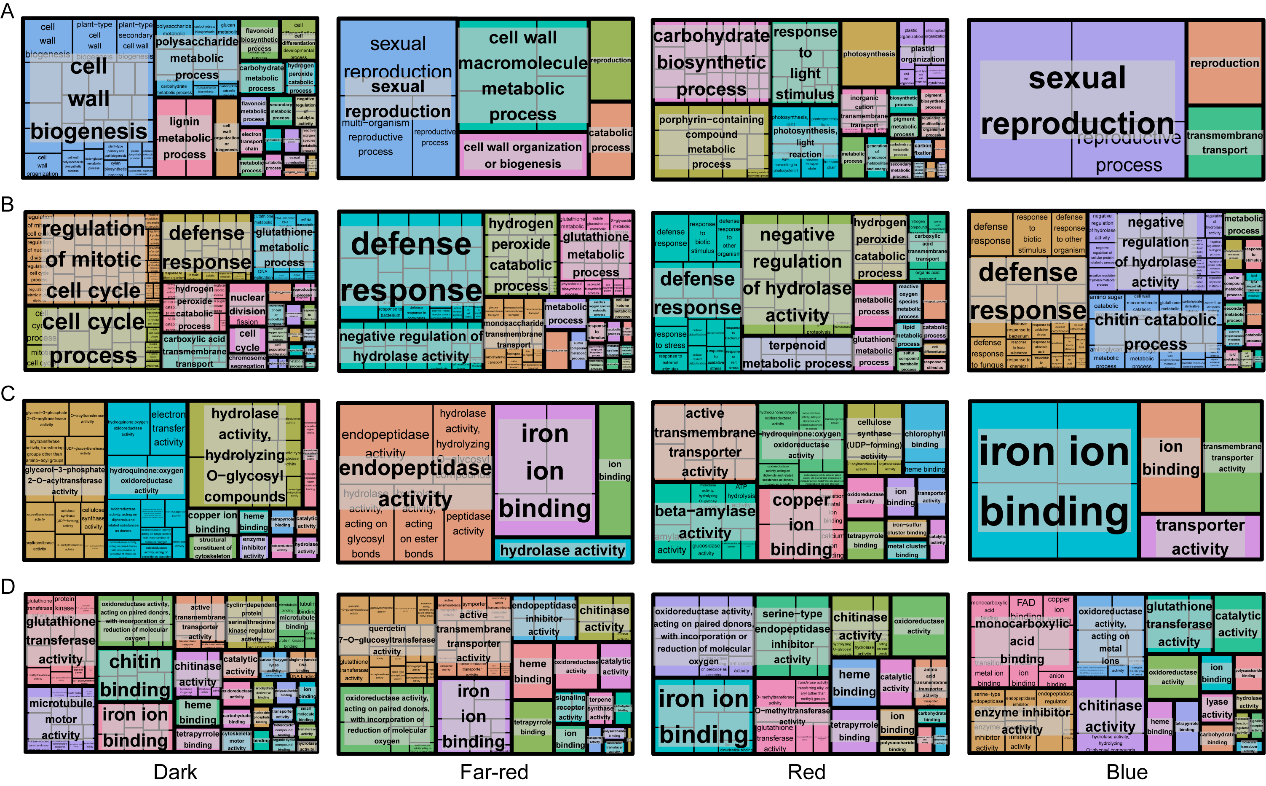


**Fig. S3.** GO enrichment categories of F_1_-MPV DEGs under various light conditions. (**A**) and (**B**) are biological process GO categories of upregulated and downregulated genes visualized using the “TreeMap” view of REVIGO, respectively. (**C**) and (**D**) are molecular function GO categories of upregulated and downregulated genes, respectively. Each rectangle indicates a single cluster representative. The representatives are shown in different colors. The size of the rectangles reflects *P*.


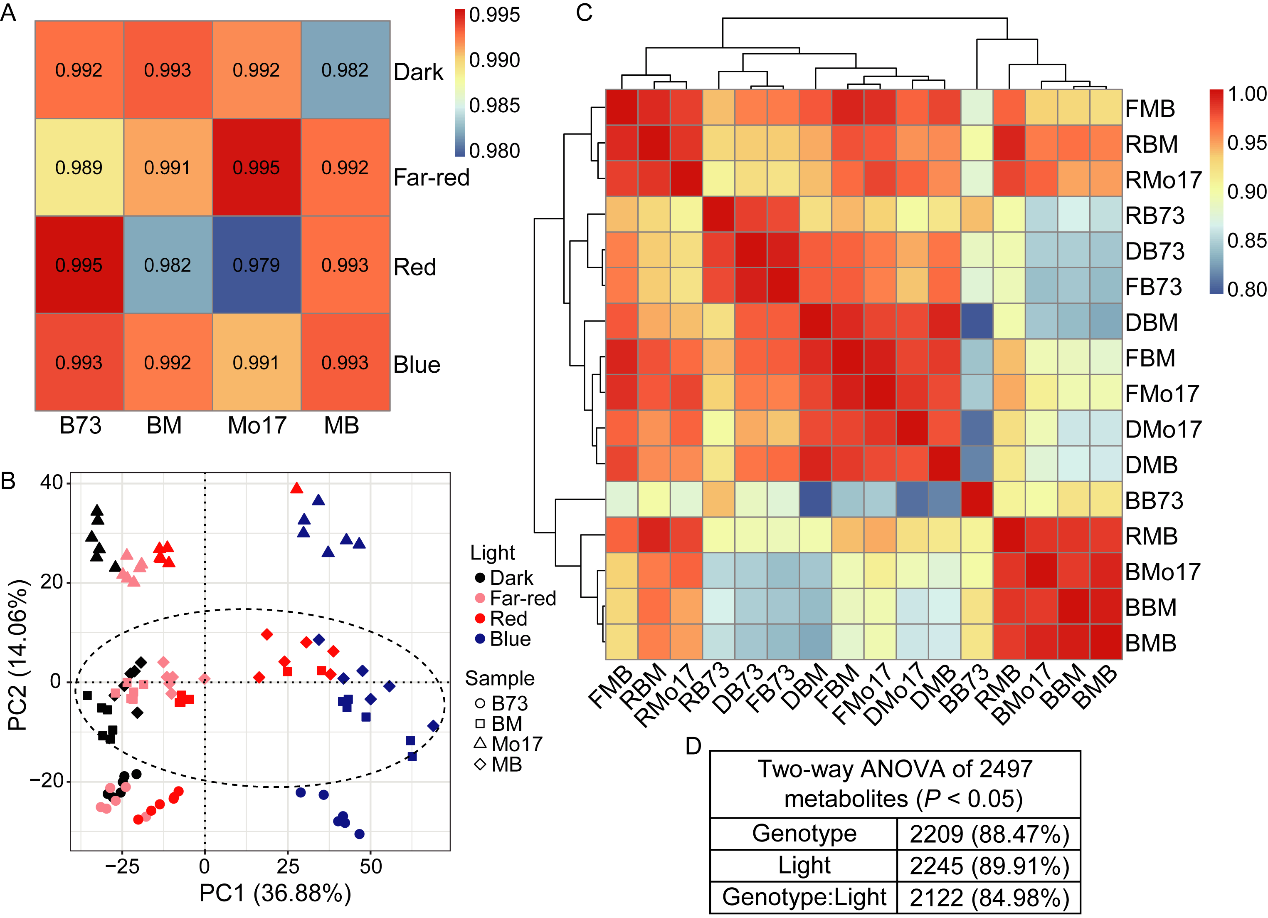


**Fig. S4.** Patterns of light and genotypic effects on maize metabolomes. (**A**) Correlation coefficients of F_1_ hybrids and parents based on metabolite accumulation under various light conditions. (**B**) PCA plot of maize metabolomes in four genotypes grown under various light conditions. (**C**) Clustering analysis of metabolite abundance profiles. The ellipses represent hybrid samples. (**D**) Two-way ANOVA of metabolites. BM and MB represent the F_1_ hybrids B73×Mo17 and Mo17×B73, respectively. DB73, DMo17, DBM, and DMB represent B73, Mo17, F_1_ hybrid BM, and F_1_ hybrid MB grown in darkness, respectively; FB73, FMo17, FBM, and FMB represent B73, Mo17, BM, and MB grown under far-red light, respectively; BB73, BMo17, BBM, and BMB represent B73, Mo17, BM, and MB grown under blue light, respectively; and RB73, RMo17, RBM, and RMB represent B73, Mo17, BM, and MB grown under red light, respectively.

**
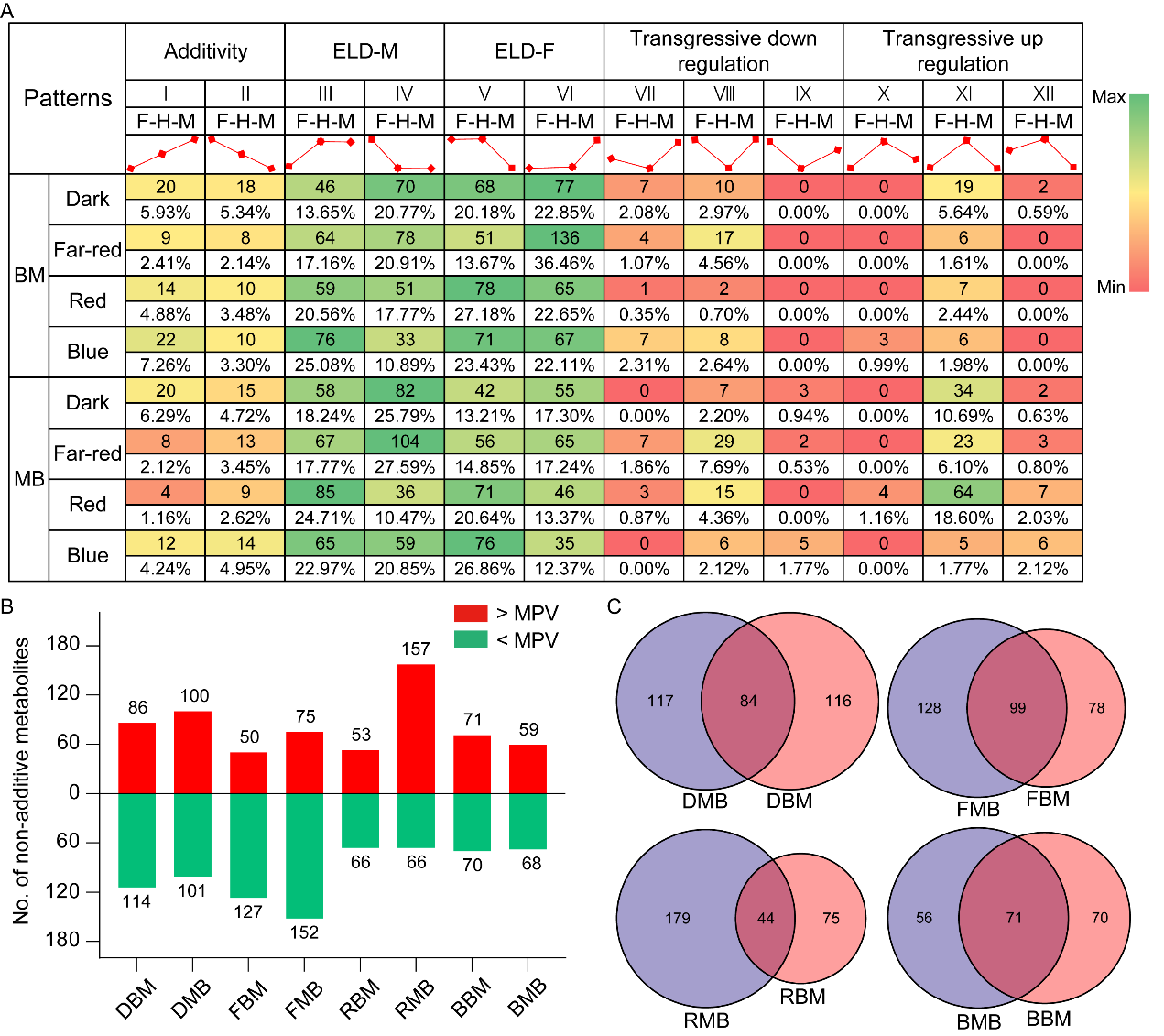
**

**Fig. S5.** Analysis of differentially accumulated metabolites in hybrids under various light conditions. (**A**) Twelve categories of differentially accumulated metabolites. VIP (variable importance in projection) > 1 and FDR < 0.05. (**B**) Number of non-additive metabolites under various light conditions. VIP > 1 and FDR < 0.05. (**C**) Overlap of non-additive metabolites between BM and MB under various light conditions. ELD-F, metabolites with accumulation similar to the female parent in the F_1_ hybrid; ELD-M, metabolites with accumulation similar to the male parent in the F_1_ hybrid. DBM and DMB represent the F_1_ hybrids BM and MB grown in darkness, respectively. FBM and FMB represent the F_1_ hybrids BM and MB grown under far-red light, respectively. RBM and RMB represent the F_1_ hybrids BM and MB grown under red light, respectively. BBM and BMB represent the F_1_ hybrids BM and MB grown under blue light, respectively.


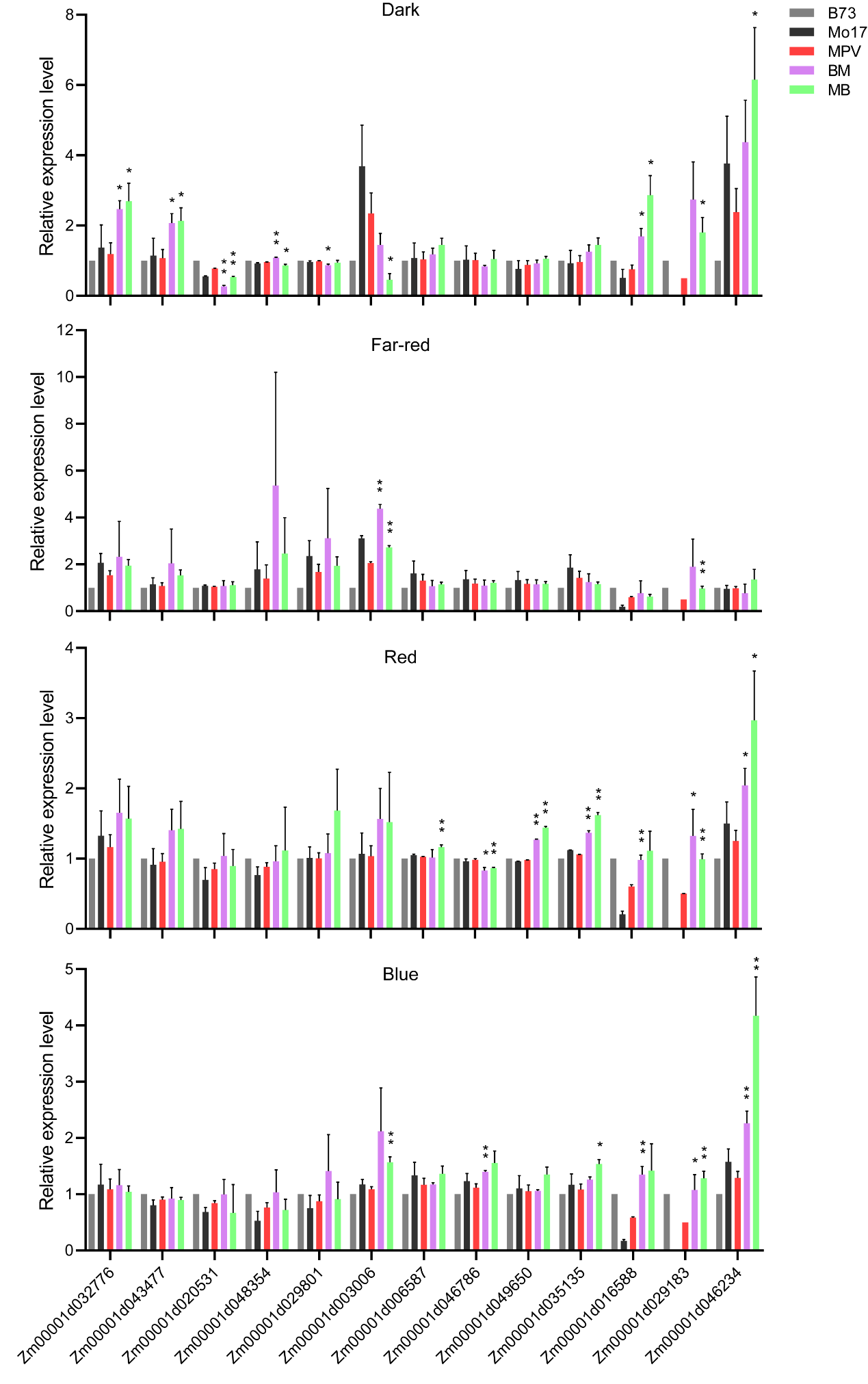


**Fig. S6.** qRT-PCR confirmation of specific DEGs under various light conditions. BM and MB represent the F_1_ hybrids B73×Mo17 and Mo17×B73, respectively. MPV represents the mid-parent value. Values are means ± standard error (SE; n = 3). * represents a significantly different expression level (*P* < 0.05) between the hybrid and MPV (Student’s *t*-test); ** represents a significantly different expression level (*P* < 0.01) between the hybrid and MPV (Student’s *t*-test).


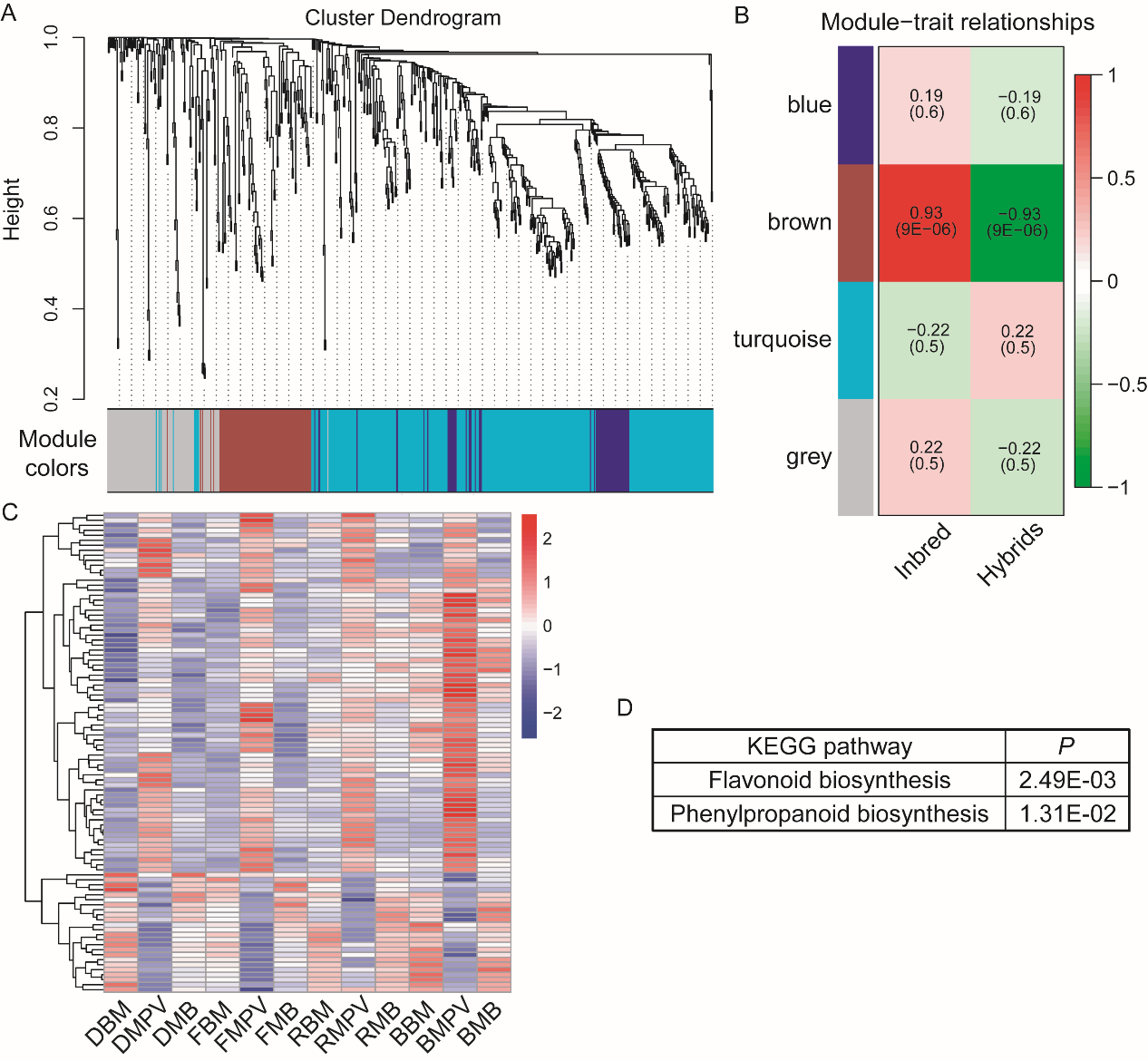


**Fig. S7.** WGCNA analysis of F_1_-MPV differentially accumulated metabolites (DAMs). (**A-B**) Co-expression networks were generated for 599 F_1_-MPV DAMs. (**C**) Clustering analysis of co-expressed metabolites in the brown module. (**D**) Table showing KEGG pathways within the brown module. The gray module represents unclustered metabolites. DBM and DMB represent the F_1_ hybrids BM and MB grown in darkness, respectively. FBM and FMB represent the F_1_ hybrids BM and MB grown under far-red light, respectively. RBM and RMB represent the F_1_ hybrids BM and MB grown under red light, respectively. BBM and BMB represent the F_1_ hybrids BM and MB grown under blue light, respectively. DMPV, FMPV, RMPV, and BMPV represent the MPV in darkness and under far-red, red, and blue light, respectively.


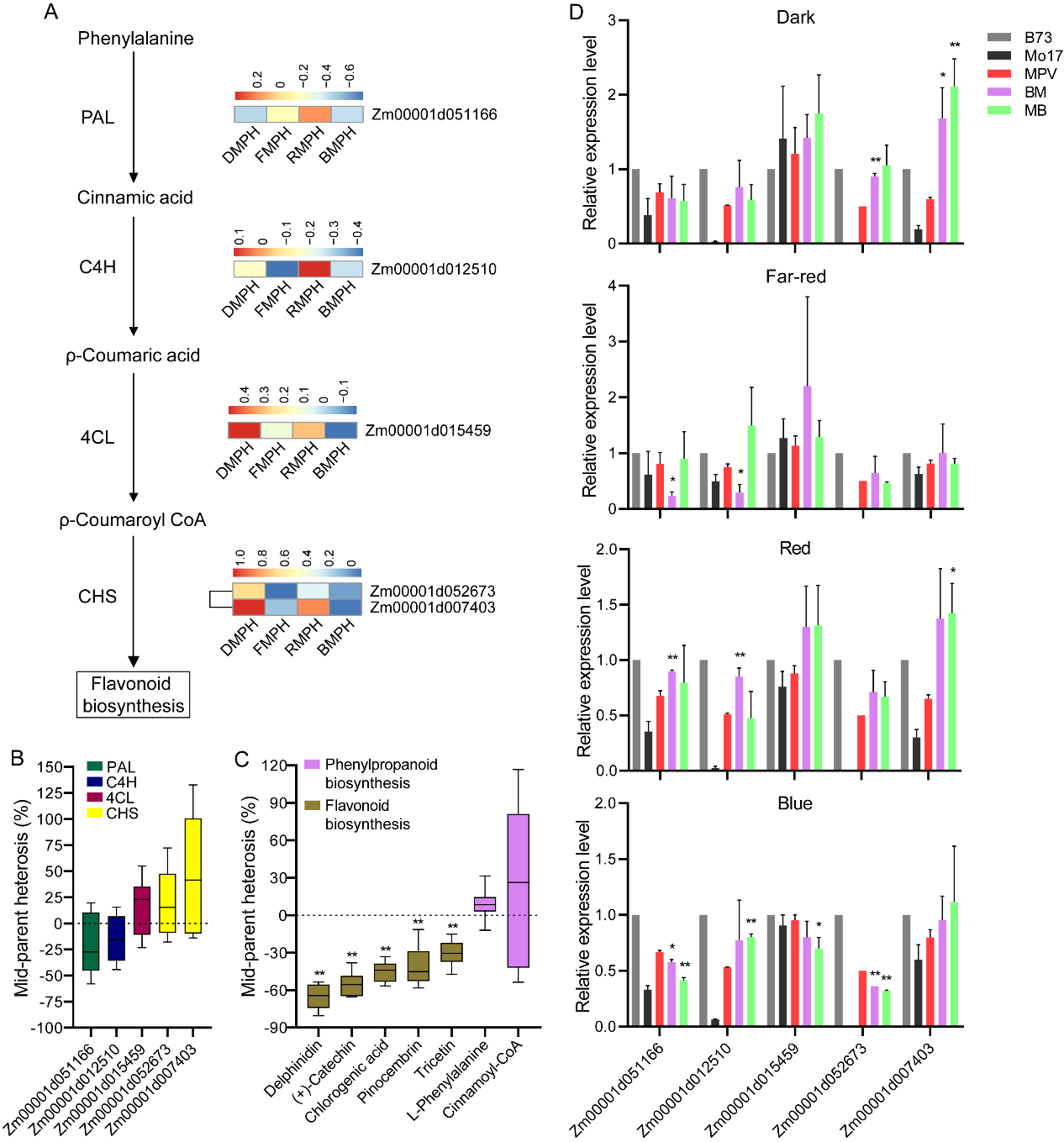


**Fig. S8.** Phenylpropanoid biosynthesis pathway under various light conditions. (**A**) Schematic diagram showing key genes and metabolites in the phenylpropanoid pathway, which was modified from Zhang and Liu [53]. Flavonoids are complex aromatic compounds synthesized through the phenylpropanoid pathway. The phenylpropanoid-flavonoid biosynthesis pathway contains several enzymes, including PAL (phenylalanine ammonia-lyase), C4H (cinnamate 4–hydroxylase), 4CL (4–coumarate-CoA ligase 1), and CHS (chalcone synthase). DMPH, FMPH, RMPH, and BMPH represent MPH in darkness and under far-red, red, and blue light conditions, respectively. (**B**) The MPH of key genes in the phenylpropanoid pathway. (**C**) MPH of key metabolites in the phenylpropanoid pathway. ** represents a significant difference at *P* < 0.01 (one sample Student’s *t*-test). (**D**) qRT-PCR confirmation of common DEGs under various light conditions. BM and MB represent the F_1_ hybrids B73×Mo17 and Mo17×B73, respectively. MPV represents the mid-parent value. Values are means ± SE (n = 3); * represents a significant expression level difference (*P* < 0.05) in the hybrid relative to the mid-parent value (Student’s *t*-test); ** represents a significant expression level difference (*P* < 0.01) in the hybrid relative to the mid-parent value (Student’s *t*-test).


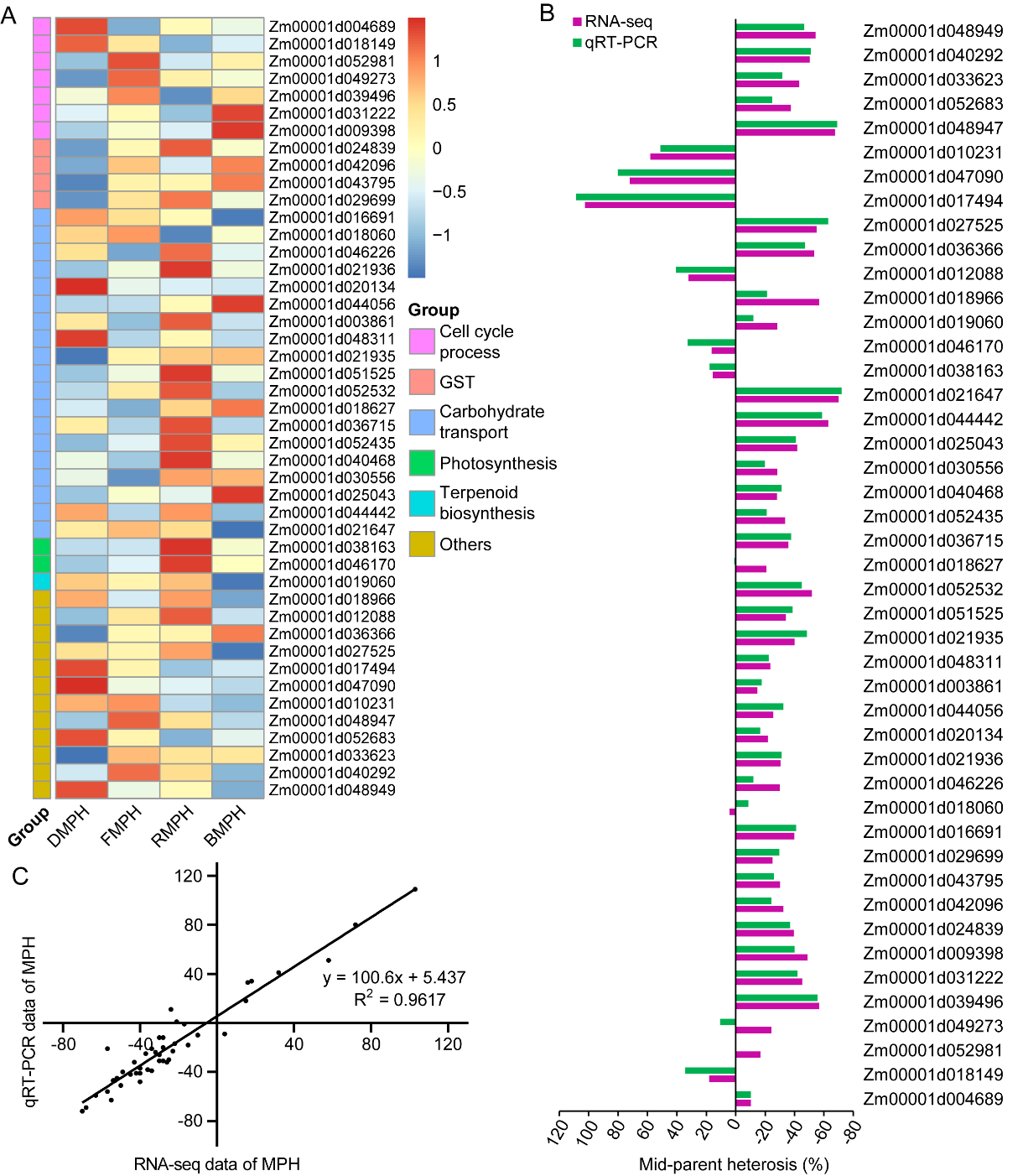


**Fig. S9.** MPH validation of 45 selected genes. (**A**) MPH of 45 selected genes based on RNA sequencing (RNA-seq) data. (**B-C**) MPH comparison between RNA-seq and qRT-PCR data for 45 selected genes.


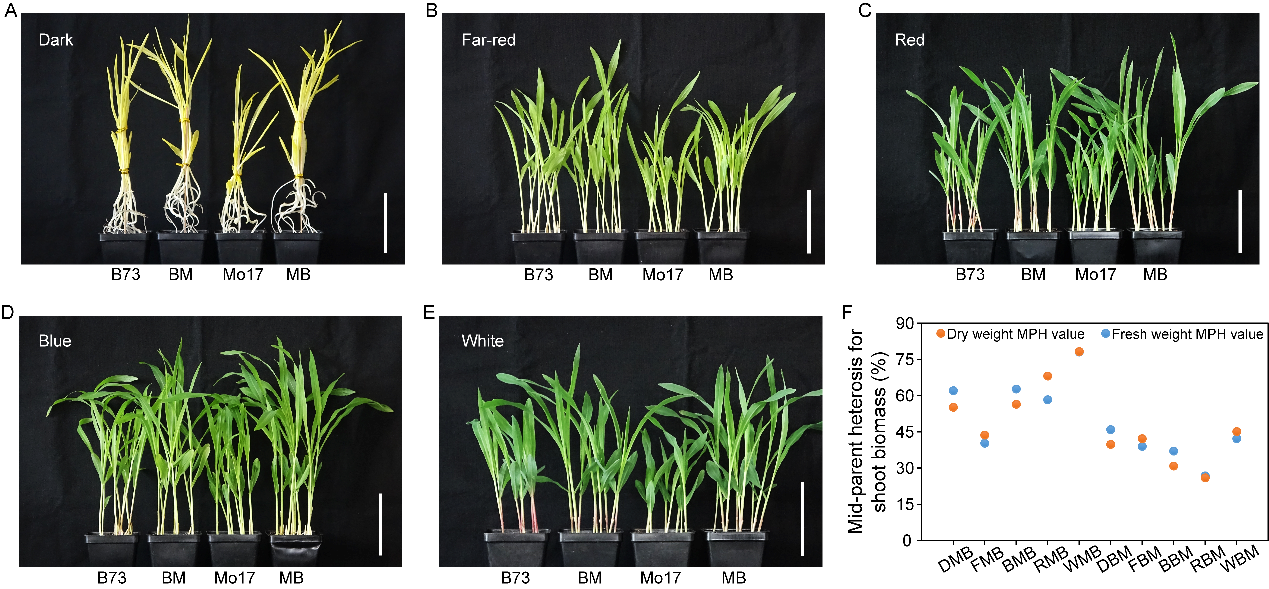


**Fig. S10.** Biomass heterosis at the seedling stage under various light conditions. (**A-E**) Seedling phenotypes of maize inbred parents and F_1_ hybrids. BM and MB represent F_1_ hybrids B73×Mo17 and Mo17×B73, respectively. The scale bar represents 10 cm. (**F**) Mid-parent heterosis of shoot biomass. DMB, FMB, BMB, RMB, and WMB represent F_1_ hybrid MB grown in darkness and under far-red, red, and blue light conditions, respectively; DBM, FBM, BBM, RBM, and WBM represent F_1_ hybrid BM grown in darkness, and under far-red, red, and blue light conditions, respectively.
